# Research on the Mechanism of “Cold Tumor” Formation and Immunotherapy for Its Transformation into “Hot Tumor”

**DOI:** 10.32604/or.2026.069317

**Published:** 2026-02-24

**Authors:** Liang Zhou, Jia Zhou, Zhengyi Wang

**Affiliations:** Department of Institute of Laboratory Animal Sciences, Sichuan Provincial People’s Hospital, School of Medicine, University of Electronic Science and Technology of China, Chengdu, 610212, China

**Keywords:** Cold tumor, immunosuppressive factors, formation mechanism, clinical treatment strategies

## Abstract

A clear goal in cold tumor research is to identify strategies for converting them into immunologically ‘hot’ tumors with enhanced immune cell infiltration and activity, thereby improving their responsiveness to immunotherapy. The genesis of cold tumors is exceedingly intricate. In recent times, as the analysis of this phenomenon has been pursued with greater depth, a suite of advanced diagnostic and therapeutic technologies has surfaced. These novel approaches and tactics are anticipated to modulate the tumor immune microenvironment across various dimensions, thereby facilitating the advancement of personalized and precise treatment modalities for cold tumors. The present article addresses the challenge of diminished therapeutic responsiveness to “cold tumors” within clinical settings. It systematically elucidates the multi-faceted regulatory mechanisms underlying immune evasion in cold tumors and offers a detailed analysis of advanced therapeutic strategies that incorporate nanotechnology, gene editing, and artificial intelligence methodologies. Furthermore, the future development trends of immunotherapy were explored in greater depth. It was posited that the convergence of artificial intelligence, multidimensional genomics, and emerging biotechnologies has presented positive prospects for the treatment of cold tumors, and has offered a theoretical foundation and technical framework for the transformation of cold tumors into “hot tumors”.

## 1 Introduction

In recent years, with the widespread application of tumor immunotherapy in clinical practice, the impact of tumor heterogeneity on its efficacy has garnered increasing attention. It is becoming increasingly important to evaluate the effects of tumor heterogeneity on the efficacy of immunotherapy and prognosis. The “hot and cold” classification and conversion strategy of clinical tumors, which are closely related to efficacy and prognosis, have gradually become the focus of immunotherapy research. Tumors are generally divided into “hot tumors” and “cold tumors” based on the intensity of the tumor immune response [1]. “Hot” tumors, also known as immune-inflammatory tumors, are characterized by a high infiltration of T cells, enhanced interferon-γ (IFN-γ) signaling, and a high tumor mutational burden (TMB). These tumors have a significant infiltration of immune cells in their microenvironment, particularly a high enrichment of effector T cells, which are in an activated state and can actively recognize and attack tumor cells. Such tumors typically exhibit high immunogenicity and can more effectively stimulate the body’s immune response. A high density of T cell infiltration is considered an indicator of a good prognosis [2]. This is common in melanoma, non-small cell lung cancer, renal cell carcinoma, and other cancers [2]. Cold tumors are further classified into two subtypes: immune-excluded tumors and immune-desert tumors. These refer to tumors with less immune cell infiltration in the tumor microenvironment (TME), tumor cells possessing a low TMB, low expression of major histocompatibility complex (MHC)-I, low immunogenicity, or strong inhibitory effects on immune cells. They also have immune suppressive cell populations and relatively low immune activity [1]. In immune-excluded tumors, CD8^+^ T cells are only located at the invasive margin and cannot effectively infiltrate the tumor. In immune-desert tumors, CD8^+^ T cells are absent from the tumor and its surrounding areas. These tumor cells are adept at evading the surveillance and attack of the immune system, making it difficult for the immune system to effectively identify and combat them. Consequently, patients with cold tumors typically do not achieve significant clinical responses when receiving immunotherapy, leading to a poor prognosis [3]. They are commonly found in cancers such as pancreatic cancer and prostate cancer [3]. In clinical practice, a low TMB cannot be entirely equated with a cold tumor phenotype. This is because the tumor immunophenotype, whether cold or hot, is not solely determined by the quantity of mutations. It is also influenced by various factors, including immunosuppressive cells, the cytokine environment, and the level of MHC expression. Moreover, certain tumor types cannot be easily categorized as either cold or hot phenotype tumors. Therefore, a comprehensive evaluation that considers both the mutation burden (genotype) and T cell infiltration/inflammatory signals (phenotype) represents a more precise clinical decision-making strategy [1,4]. Refer to Table 1 for controls of the cold and hot tumor microenvironments. In clinical immunotherapy strategies targeting cold tumors, it is particularly important to explore strategies to convert cold tumors into hot tumors. However, the formation mechanisms of cold tumors are complex and diverse; thus, a thorough understanding of their pathogenesis is crucial for developing effective treatment strategies.

**Table 1:** Comparison table of TME between cold tumors and hot tumors

Dimension	Cold tumor (“immune desert” type)	Hot tumor (“inflammatory” type)	Reference
Core definition	Low immune cell infiltration, extremely weak immune response	High immune cell infiltration, presence of activated anti-tumor immunity	[1–4]
T Cell infiltration	Very few CD8^+^ CTLs, sparse Tumor infiltrating lymphocytes (TILs); Regulatory T cells (Tregs) and Myeloid-Derived Suppressor Cells (MDSCs) are relatively dominant	Abundant CD8^+^ CTLs, effector T cells; dense TILs	[1–3]
Helper immune cells	Insufficient or dysfunctional dendritic cells (DCs) and Natural Killer Cells (NK cells)	Mature DCs, significantly increased activated NK cells	[1,2]
Myeloid cell lineage	M2-type tumor-associated macrophages (TAMs) and MDSCs are predominant, promoting immune suppression	M1-type TAMs and inflammatory monocytes predominate, secreting pro-inflammatory factors	[1,2]
Immune checkpoints	Low or absent PD-L1 expression; lack of IFN-γ signaling	High PD-L1 expression; IFN-γ signaling active, responsive to Immune checkpoint inhibitors (ICI)	[1,2,4]
Vascular characteristics	Highly abnormal vasculature: twisted, leaky, irregular basement membrane, insufficient pericyte coverage; leading to low perfusion, hypoxia, acidosis	Relatively normalized vasculature: basement membrane regular, endothelial cell junctions tight, pericyte coverage good; adequate perfusion and oxygen supply	[1,4]
Metabolic microenvironment	Lactic acid accumulation, low pH, high osmotic pressure; depletion of glucose and amino acids, inhibiting T cell function	Relatively normal pH and oxygen tension; abundant metabolite supply, conducive to immune cell survival and function	[1–3]
Tertiary Lymphoid Structures (TLS)	Rare or absent	Common and mature, including B cell follicles and germinal centers, supporting sustained immune response	[1,3]
Prognosis and Immunotherapy response	Poor response to ICI, requires combined strategies to ‘heat up’	Good response to ICI, relatively good prognosis	[1–4]

## 2 Mechanism of Cold Tumor Formation

### 2.1 Cold Tumor Formation Classical Cognition

Tumors are genetically related diseases [5]. Under the influence of various predisposing factors—including aging, chemical exposure, physical factors, viral infection, lifestyle, immune system disorders, and genetic susceptibility—normal cells in the human body may exhibit pathological changes, such as dysfunction or gene mutations. These changes result in abnormal mutations in somatic cells via various mechanisms, including genomic instability, epigenetic alterations, and disruptions in cell cycle regulation. These mutated cells undergo a complex, multi-step process that involves key stages such as clonal expansion, immune evasion, microenvironment remodeling, and angiogenesis, ultimately leading to the formation of solid tumors with heterogeneous characteristics. The development of cold tumors is a multi-level, gradual evolutionary process [3,5].

At the internal level, a major factor in the formation of cold tumors is the reduced immunogenicity of their tumor cells. During the early stages of tumor development, these variant cells frequently express tumor-specific antigens (TSA) or tumor-associated antigens (TAAs). However, as the tumor progresses, these heterogeneous cells can evade recognition by CD8^+^ T cells by down-regulating MHC-I molecules, through β2-microglobulin mutations, or due to defects in the transporter associated with antigen processing (TAP), resulting in an inability to present TSA and TAA [6,7]. Certain solid tumors, such as those affecting the pancreas and prostate, may lead to the loss of immunogenic neoantigens due to a low mutational burden or clonal selection pressure [8,9]. During the process of evading immune system surveillance, tumor cells not only downregulate antigen expression but also markedly enhance the expression of immune checkpoint ligands, such as PD-L1, on their cell membranes [10]. Additionally, they secrete inhibitory cytokines, including transforming growth factor β (TGF-β) and interleukin-10 (IL-10), further diminishing the immunogenicity of the tumor [11].

From an external perspective, the development of cold tumors is closely associated with the isolation effect resulting from physical barriers. In TME, tumor-associated fibroblasts (CAFs) can synthesize and remodel the extracellular matrix, including components like collagen and fibronectin, thereby increasing matrix stiffness and limiting the infiltration of effector T (Teff) cells. This matrix remodeling serves as a physical barrier to prevent direct contact between immune cells and tumor cells [12]. Furthermore, both tumor cells and CAFs secrete a diverse array of angiogenic factors, including vascular endothelial growth factor (VEGF), angiopoietin (Ang), and fibroblast growth factor (FGF), which collectively facilitate incomplete tumor angiogenesis [13]. Tumor vessels typically exhibit irregular structural characteristics, lack mature pericytes and basement membrane support, and their walls appear weak and unstable. The specific manifestations include increased permeability of the vessel walls, abnormal proliferation of vascular endothelial cells, and an incomplete angiogenic process, which collectively result in vessels that are prone to collapse and blood flow interruption [14]. These abnormal immature tumor blood vessels are unable to effectively deliver immune cells to the inside of the tumor tissue, forming an “immune barrier”. The high permeability of tumor blood vessels leads to leakage of plasma and proteins into the surrounding interstitial space, increasing interstitial fluid pressure and further hindering blood perfusion, resulting in the difficulty of effective infiltration of peripheral immune cells into the interior of the tumor tissue, thus forming a cold tumor physical environmental barrier [13,15].

At the cellular level, it is crucial to construct a cold tumor immunosuppressive microenvironment. The suppressive cytokines secreted by tumor cells can attract immunosuppressive cells, such as tumor-associated macrophages (TAMs), regulatory T cells (Tregs), and myeloid-derived suppressor cells (MDSCs). These cytokines also activate the functions of these immunosuppressive cells, thereby further contributing to the suppression of the tumor immune response [11]. The tumor continued to expand due to competitive growth following the metabolic reprogramming of tumor cells. Moreover, the scarcity of metabolic substrates and transport circulation support led to the accumulation of lactic acid, adenosine, and other substances in the tumor microenvironment, as well as the depletion of arginine. These factors further inhibit T cell activity and promote the formation of a hypoxia-high interstitial pressure immunosuppressive microenvironment. Hypoxia has the capability to enhance α factor (HIF). HIF-1α and HIF-2α regulate the adaptive response of tumor cells via complex interactions with the PI3K/AKT/mTOR, MAPK/ERK, and NF-κB signaling pathways. Under acute hypoxia, HIF-1α is rapidly stabilized and activated, and subsequently translocated to the nucleus where it binds to the hypoxia response element (HRE) to induce the expression of target genes. The text includes genes associated with glycolysis and lactic dehydrogenase. By upregulating genes involved in glycolytic metabolism, the entry of pyruvate into mitochondria is inhibited, thereby encouraging aerobic glycolysis [16]. Concurrently, this process is frequently accompanied by the inactivation of tumor suppressor genes, such as P53, which alleviates their inhibitory effects on glycolysis, for example, through the downregulation of TIGAR. This also promotes oxidative phosphorylation, as seen in the downregulation of SCO2, thus shifting energy metabolism towards aerobic glycolysis. The activation of oncogenes can also lead to the up-regulation of glycolytic enzymes. Under the combined influence of multiple factors, the aerobic glycolysis metabolism in tumor cells is further enhanced, resulting in the production of a large amount of lactic acid [16–18]. Hypoxia can also result in dysfunction of the mitochondrial electron transport chain (ETC) and an increase in reactive oxygen species (ROS) production. This, in turn, promotes mutations in mitochondrial DNA and exacerbates metabolic disorders and tumor progression [19]. This disordered metabolic reprogramming, referred to as the Warburg effect, ultimately leads to acidification of the microenvironment. These metabolic alterations not only impede the function of immune cells but also promote the survival and function of certain immunosuppressive cells. For instance, a hypoxic environment significantly enhances the function of CAFs by upregulating and activating transcription factors such as HIF-1α and nuclear factor E2-related factor 2 (NRF2), endowing them with a stronger cancer-promoting ability [20]. These metabolic alterations not only impede the function of immune cells but also promote the survival and function of certain immunosuppressive cells. For instance, a hypoxic environment significantly enhances the function of CAFs by upregulating and activating transcription factors such as HIF-1αand NRF2, endowing them with a stronger cancer-promoting ability [20]. Specifically, CAFs can regulate the local metabolic environment by inducing indoleamine-2,3-dioxygenase (IDO), which generates immunosuppressive metabolites like adenosine. This process more effectively inhibits the function of immune cells within the TME, leading to their functional failure [12]. Under the synergistic action of these multiple cells, a tumor microenvironment with unique immunosuppressive effects is formed and stabilized, constituting the basic condition for the formation of cold tumors.

From the molecular-level analysis of tumors, epigenetic mechanisms and signal pathway regulations are pivotal in the formation of cold tumors. Epigenetics plays a crucial role in the mechanism of cold tumor formation. Epigenetic modifications (e.g., DNA methylation) can alter the expression of critical genes. Specifically, DNA methyltransferase overexpression induces hypermethylation of tumor suppressor gene promoters, suppressing their activity and facilitating tumor progression [21]. High expression of histone deacetylase disrupts the regulation of tumor-related genes, affecting the processes of cell proliferation, differentiation, and apoptosis, thereby increasing the malignancy of tumor cells [22]. Epigenetic modifications can not only impair antigen presentation by downregulating MHC-I expression or function but also affect the expression of other immune-related molecules within tumor cells, such as interferon receptors and costimulatory molecules. These effects further diminish the immunogenicity of tumor cells, modulate the function and infiltration of immunosuppressive cells, inhibit immune effector cells, and impact the communication between tumor cells and other cells in the tumor microenvironment, thereby reshaping the immune microenvironment. lncRNA and miRNA also play a significant role in regulating epigenetic modifications. Numerous lncRNAs are involved in regulating the M1/M2 polarization of macrophages, which in turn affects the tumor microenvironment [23,24]. For example, ANCR can reduce the concentration of M1 macrophage marker molecules IL-1β and IL-6 in macrophage supernatant and inhibit the M1 polarization of macrophages [23,24]; LINC00662 further promoted the polarization of M2 macrophages through paracrine activation of the Wnt/β-catenin signaling pathway in macrophages [25]. The expression of miR-155 is up-regulated in various types of tumors. It can regulate the maturation and function of Dendritic cells (DCs), and impact the activation of T cells and immune responses. In cold tumors, the aberrant expression of miR-155 may suppress the function of immune cells and facilitate tumor immune evasion [26]. The PI3K/AKT/mTOR, MAPK/ERK, and NF-κB signaling pathways are activated by the combined effects of tumor suppressor gene *PTEN* loss and factors such as TGF-β and IL-6. The continuous activation of these three oncogenic signaling pathways is an important molecular basis for the formation of “cold tumors”. Through the two-way regulatory mechanism of the “tumor cell-immune microenvironment,” they systematically weaken the anti-tumor immune response, inhibit the infiltration and function of CD8^+^ T cells, and thereby construct an immunosuppressive microenvironment. For instance, the activation of the PI3K/AKT pathway can upregulate PD-L1, decrease the expression of antigen-presenting molecules (MHC-I), inhibit the maturation of dendritic cells, and prevent CD8^+^ T cells from infiltrating the tumor parenchyma [27,28]. Simultaneously, it markedly elevated the protein and mRNA levels of HIF-1α, resulting in its accumulation in the nucleus and enabling it to function effectively as a transcription factor.

Furthermore, from the perspective of the human body as a whole, the development of cold tumors is often accompanied by systemic immunosuppression. Tumors interact with the systemic immune system. Cold tumor cells evade recognition and attack by the immune system through various mechanisms, such as the upregulation of PD-L1 and the downregulation of MHC-I expression. These mechanisms influence not only the local tumor microenvironment but also the systemic immune system. The dysfunction of the entire body’s immune system will further feed back into the tumor microenvironment, exacerbating immunosuppression and creating a vicious cycle. For instance, the secretion of G-CSF by tumors results in bone marrow generation that favors immature neutrophils (PMN-MDSCs), thereby weakening the body’s immune surveillance [29]. Meanwhile, neuro-endocrine regulation, such as chronic stress (β-adrenergic signaling), promotes tumor metastasis and suppresses Natural Killer (NK) Cell function [30].

**Figure 1:**
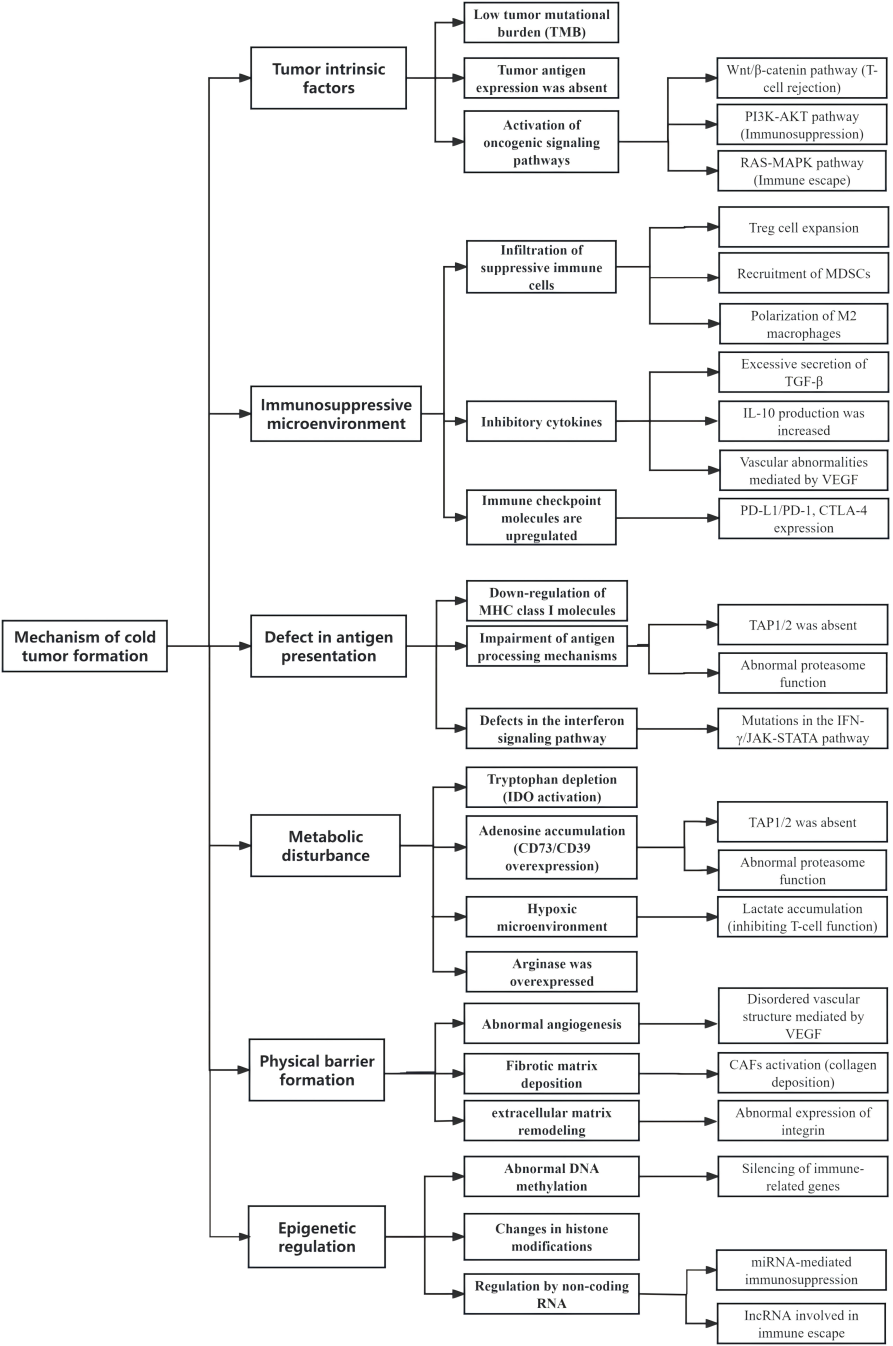
Summary of the mechanisms of cold tumor formation

**Figure 2:**
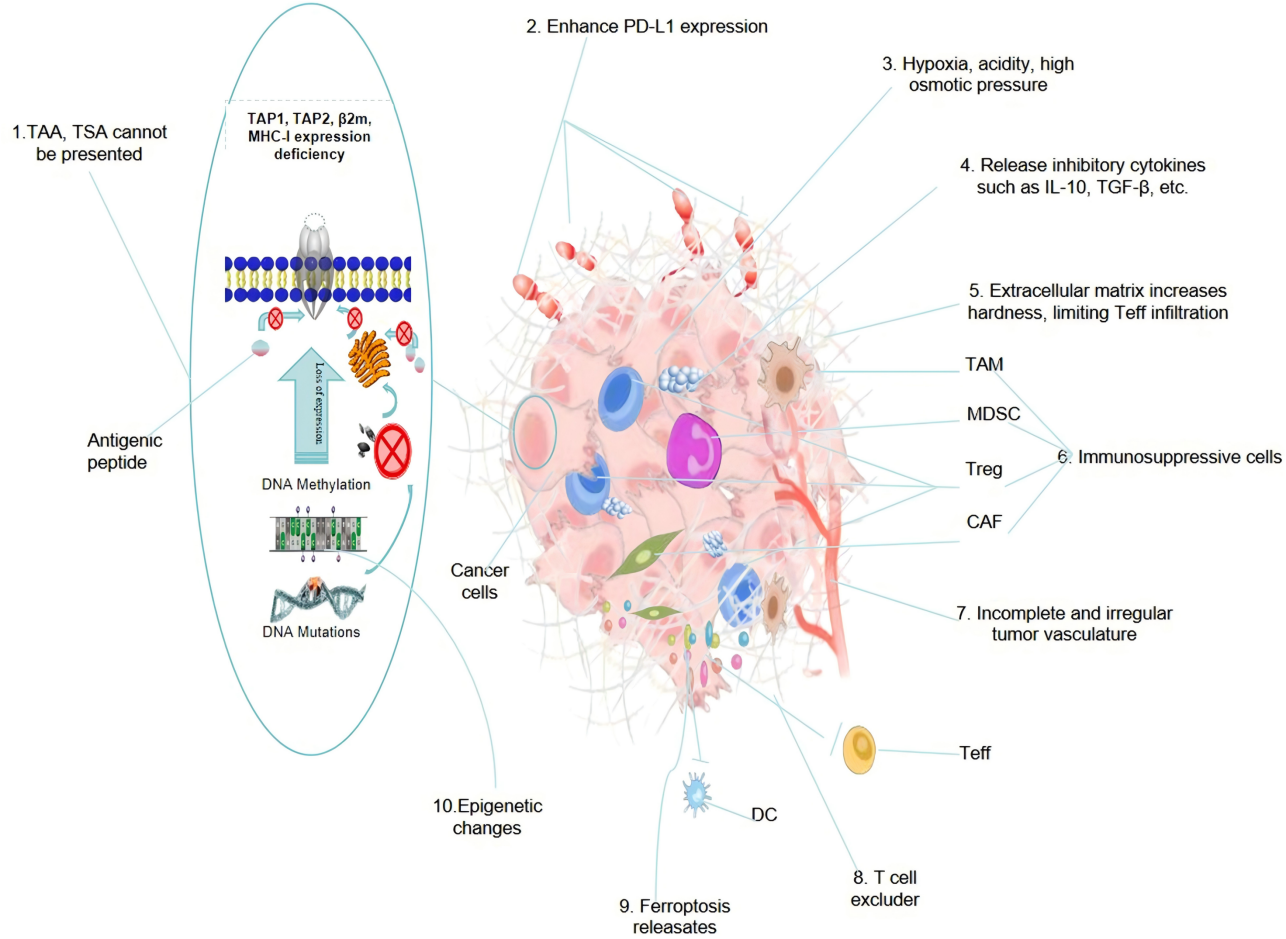
A summary diagram of the mechanism of cold tumor formation

In summary, the formation of cold tumors is a complex process that involves the characteristics of the tumor cells themselves, alterations in the tumor microenvironment, and dynamic changes in the immune system. This multifaceted process encompasses cellular, molecular, and immunological microenvironmental aspects, representing a gradual evolutionary progression from an initially immunogenic state to an immunosuppressive state. For a comprehensive overview of the classic mechanisms underlying cold tumor formation, please refer to Figs. 1 and 2.

Current research has revealed the complexity, diversity, and heterogeneity of the mechanism of cold tumor formation, with different individuals potentially driven by different dominant factors. The diagram above summarizes some findings from modern research across 10 aspects, specifically including: 1. Defects in tumor antigen presentation lead to TAA and TSA not being presented to the cell surface via the MHC-1 (HLA-1) protein complex, preventing Antigen presenting cell (APC) from presenting and activating T cells, which in turn cannot be recognized by T cells, resulting in immune evasion. 2. Tumor cells, to evade attacks from immune cells, autonomously regulate the generation of immune checkpoint ligands (such as PD-L1), which, by binding to the effector cell receptor (PD-1), deplete immune effector cells. 3. Hypoxia, accumulation of acidic metabolites (Warburg effect), and immune metabolic reprogramming within the TME lead to functional exhaustion of Teff, and the high local osmotic pressure makes it difficult for immune-active cells to penetrate the tumor, but under the action of specific cytokines, it is more conducive to the activity and infiltration of immune-suppressive cells. 4. A large number of immune-suppressive factors generated by immune-suppressive cells exist within the TME, which can rapidly deplete immune-active cells, reducing the number and infiltration ability of pro-inflammatory immune cells within the tumor. 5. Activation of cancer-associated fibroblasts (CAF) remodels the extracellular matrix, making the tumor extracellular matrix denser and harder, making it difficult for peripheral immune cells to infiltrate. 6. Immune-suppressive factors can attract immune-suppressive cells, including TAMs, Tregs, MDSCs, and CAF, and can activate the functions of these cells, thereby promoting the suppression of anti-tumor immune responses. 7. Irregularly structured tumor blood vessels lack mature pericytes and basement membranes, have thin and unstable vessel walls, increased permeability, abnormal proliferation of vascular endothelial cells, and incomplete angiogenesis, leading to vessel collapse and blood flow interruption, making it difficult for immune cells to infiltrate. 8. Recent research has found that the tumor extracellular matrix may be a key factor causing difficulty for T cells to infiltrate into tumors in cold tumors, with tumor extracellular matrix being abundantly secreted in tumors, strongly repelling T cells. 9. Cell ferroptosis has a dual role in promoting and inhibiting immunity, and under certain conditions, substances released such as 4-hydroxynonenal (4-HNE), hinder the maturation of DC and damage their ability to present antigens, reducing anti-tumor immune responses, thereby promoting the formation of cold tumors instead. 10. Epigenetic changes such as DNA methylation, histone modification, and non-coding RNA regulation can affect the expression of genes related to immune responses and tumor antigen presentation, thereby promoting the formation of cold tumors. Abbreviations: TAAs (Tumor associated antigens); TSA (Tumor specific antigen); TAP1/2 (Transporter associated with antigen processing1/2); TAM (Tumor associated macrophage); MDSCs (Myeloid-derived suppressor cells); Tregs (Regulatory T cells); Teff (effector T) cells; CAF (Tumor associated fibroblast); DC (Dendritic cell); β2m (Beta-2 Microglobulin); MHC-Ⅰ (Major Histocompatibility Complex Class I); PD-1 (Programmed Cell Death Protein 1).

### 2.2 Research on the Mechanism of Cold Tumor Formation Is in Progress

Observations from classical studies reveal that significant progress has been made in innovative research on the mechanism of cold tumor formation worldwide in recent years. Building upon the insights and findings of previous studies, global research has achieved various breakthroughs in understanding the mechanism of cold tumor formation, resulting in the construction of a relatively complete mechanism system. These advancements encompass in-depth research into the critical question of how cold tumors and tertiary lymphoid structures influence disease prognosis, as well as the application of metabolomics to understand the metabolic characteristics of cold tumors. Additionally, the use of artificial intelligence (AI) to comprehensively analyze multi-dimensional omics data and explore potential therapeutic targets is included. These advances offer new perspectives and methods for the diagnosis and treatment of cold tumors in the future and suggest that further breakthroughs in this field are anticipated.

#### 2.2.1 Cold Tumors and Tertiary Lymphoid Structures (TLS)

TLS does not exist in human tissues under normal physiological conditions. It is caused by pathological conditions such as chronic inflammation or tumors, which trigger a series of complex molecular and cellular signaling pathway changes, disrupt the normal homeostasis of tissues, and lead to the formation of ectopic lymphoid structures in non-lymphoid tissues [31]. TLS shares structural features with secondary lymphoid organs, such as lymph nodes, typically including germinal centers, T cell zones, B cell zones, and high endothelial venules. However, its organizational structure is relatively looser and less mature compared to that of secondary lymphoid organs [32]. In the tumor tissue, TLS consists of B cells, T cells, DC, high endothelial venules (HEV), and a network of fibroblasts, encompassing both T cell areas and follicular B cell areas [33]. In TLS, HEVs serve a critical role as a “transportation hub.” They possess a unique structure where endothelial cells express adhesion molecules, including lymphocyte homing receptor ligands such as Mad-CAM-1, which guide lymphocytes (both B cells and T cells) from the circulation to the TLS. The presence and function of HEVs are crucial for the dynamic renewal of immune cells within the TLS and for maintaining an appropriate number of immune cells in the TLS [34]. Researchers believe that the formation of TLS may be initiated by antigen stimulation within the TME. In addition to the chemotactic role of Mad-CAM-1, chemokines such as CXCL13 and CCL21 secreted by various cells within the TME also serve as “road markers” to guide immune cells to accumulate in the tumor tissue. For instance, CXCR5-positive B and T cells are able to migrate into the tumor along a concentration gradient of CXCL13 [35]. Upon local aggregation, immune cells, particularly T cells, become fully activated in response to antigen stimulation and costimulatory signals. Activated T cells then secrete a range of cytokines, including IL-2 and IFN-γ. These cytokines, on the one hand, can enhance the activity of other immune cells, such as additional T cells and natural killer cells, thereby further amplifying the immune response; On the other hand, they can also exert a direct inhibitory effect on tumor cells, such as IFN-γ, which can inhibit tumor cell proliferation and angiogenesis [36,37]. B cells experience somatic hypermutation and undergo antibody affinity maturation in response to antigen stimulation and with the assistance of T cells. They are capable of producing high-affinity antibodies directed against tumor antigens, which can bind to antigens on the surface of tumor cells and eliminate them by activating the complement system or through antibody-dependent cell-mediated cytotoxicity (ADCC) [38]. The activation process of these immune cells is the key force behind the formation of TLS. As immune cells continuously accumulate and become activated, they interact with one another and begin to construct the cellular network structure of TLS, thereby forming a relatively mature and organized tertiary lymphoid structure. This structure plays a more effective local immune defense role and continuously attacks and monitors tumor cells [39]. In the field of medical research, TLS has been identified in various types of tumors, including lung cancer, breast cancer, and melanoma, among others [33]. The occurrence of TLS is often closely related to a better prognosis and a positive response to immunotherapy. The presence and maturity of TLS can serve as prognostic markers for certain diseases [40]. For instance, in various types of cancer, the density and maturity of intratumoral TLS are closely correlated with the patient’s survival time, treatment response, and other prognostic indicators, aiding doctors in evaluating the patient’s condition and prognosis [41].

In cold tumors, a series of intrinsic and extrinsic factors hinder the formation of TLS. These factors are intertwined and interact with each other, making the effective formation of TLS in cold tumors difficult. The overall status of the host immune system plays a crucial role in the formation of TLS within cold tumors. Cold tumor hosts are typically in a state of immunosuppression, often due to long-term radiotherapy and chemotherapy, immunodeficiency diseases, or the use of immunosuppressive drugs. This makes it challenging to form effective TLS even in the presence of tumors [42]. There is a significant presence of immunosuppressive cells, such as Treg and MDSC, within cold tumors. These cells secrete immunosuppressive factors, including TGF-β and IL-10, which inhibit the activity and proliferation of immune effector cells, thereby impeding the formation of TLS. Additionally, the reduced expression or imbalance of certain cytokines, such as IL-6 and IL-21, and chemokines like CXCL13 and CCL21, which are responsible for guiding immune cells to cluster at tumor sites, can also hinder the construction of TLS [35]. Cold tumor cells inherently possess low immunogenicity or the ability to evade the immune system. They express fewer antigens or produce immunosuppressive molecules, such as PD-L1, CTLA-4 ligand, CD73, and IDO, which complicates the immune system’s recognition and attack on tumor cells and hampers the formation of TLS [3]. The accumulation of tumor cell metabolites, such as lactic, can alter the local microenvironment, suppress immune cell function, and impede the formation of TLS [43]. Due to the plasticity of effector immune cells, they experience functional exhaustion in the cold tumor microenvironment. This affects the infiltration and organization of immune cells in cold tumors, thereby hindering the formation of TLS [44]. Similar to hot tumors, cold tumors also exhibit significant heterogeneity, with tumor cells in various regions displaying diverse gene expression, phenotypes, and functions. Despite the seemingly inconsistent tumor response to immune responses due to this heterogeneity, generally, all regions of the cold tumor manifest a relatively uniform state of immunosuppression, which hampers the formation of cold tumor TLS [1]. Additionally, the abnormal structure of tumor blood vessels and the accumulation of matrix components create a physical barrier that hinders immune cells from infiltrating the tumor, thus presenting obstacles to the generation of TLS [45].

In conclusion, intrinsic factors that hinder the formation of TLS in cold tumors encompass characteristics of tumor cells, expression of immunosuppressive molecules, and metabolic characteristics. Extrinsic factors include inadequate infiltration of immune cells, the presence of immunosuppressive cells, and specific cytokines such as IL-7, IL-15, IL-21, and CXCL13, which are required for the formation of TLS in the cytokine environment. These adverse factors intertwine and interact, creating a vicious cycle that permeates various stages of TLS formation, such as the recruitment, migration, activation, and proliferation of immune cells, as well as the maturation and maintenance of TLS. In cold tumors, the disorder in TLS formation results in a compromised tumor immune microenvironment, reduced treatment efficacy, a higher risk of recurrence and metastasis, a lower survival rate, and a poor prognosis. On the other hand, if we can intervene and regulate these factors using innovative methods, it is anticipated that we can promote the formation of TLS in cold tumors, thereby improving the efficacy of immunotherapy.

#### 2.2.2 Metabolomics and Cold Tumors

Metabolomics is a scientific discipline that concentrates on the study of all metabolites and their alterations within organisms. In recent years, it has been extensively applied in cancer research. During the growth, proliferation, and invasion of tumor cells, they exhibit a comprehensive and complex phenomenon of metabolic reprogramming that is markedly different from that of normal cells. This metabolic reprogramming not only aids tumor cells in adapting to the harsh microenvironment but also holds significant implications for tumor development and treatment.

There are numerous differences in metabolomics between cold tumors and hot tumors, reflecting the distinct characteristics of these tumor types in energy metabolism and immune microenvironment, among other aspects. These differences are outlined below: A. Glucose metabolism. The Warburg effect in tumor cells is described in Section 2.1. Due to the reduced infiltration of immune cells in cold tumors, the uptake and utilization of glucose by these tumor cells is relatively more pronounced. Consequently, cold tumors often exhibit a higher rate of glycolysis. B. Fatty Acid Metabolism. Cold tumor cells exhibit increased uptake and oxidation of fatty acids. Within the hypoxic tumor microenvironment, these cells utilize fatty acid oxidation to generate energy by up-regulating proteins associated with fatty acid uptake. Additionally, fatty acid metabolites can function as signaling molecules, regulating the proliferation and survival of tumor cells [46]. Fatty acid utilization by immune cells is relatively high in hot tumors, which may limit fatty acid metabolism in tumor cells. Cold tumors and hot tumors exhibit different characteristics in the composition and properties of fatty acids. Cold tumors may contain a higher proportion of unsaturated fatty acids, which can alter the fluidity and function of the cell membrane, thereby affecting the signaling and immune escape ability of tumor cells. In contrast, the fatty acid composition of hot tumors is more “healthy,” which is more conducive to the normal function of immune cells [47]. C. Amino Acid Metabolism. Glutamine Metabolism: Glutamine metabolism is pivotal as it serves as a key energy source and biosynthetic precursor for tumor cells. Typically, cold tumor cells exhibit high expression of glutaminase, enabling them to uptake and metabolize glutamine in substantial amounts to support their rapid proliferation. This aberrant glutamine metabolic activity disrupts the amino acid equilibrium within the tumor microenvironment, subsequently impacting the function of immune cells. In contrast, hot tumors have tumor cells that are less reliant on glutamine, permitting immune cells to utilize glutamine more effectively to sustain their activity and function [48]. Beyond abnormal glutamine metabolism, cold tumors also exhibit other amino acid metabolism disorders, including imbalances in arginine and tryptophan metabolism. Regulatory T cells deplete arginine by increasing arginase activity, thereby suppressing the function of effector T cells and enhancing the immunosuppressive microenvironment. In contrast, such occurrences are less common in hot tumors, where the amino acid microenvironment surrounding immune cells is more conducive to their immune surveillance and cytotoxic functions [49]. Key enzymes such as IDO1 and tryptophan 2,3-dioxygenase (TDO), which are responsible for catalyzing the conversion of tryptophan to kynurenine (Kyn) and other metabolites, are upregulated in a variety of cold tumors. Kyn and its derivatives can bind to aryl hydrocarbon receptor (AhR) and activate AhR signaling pathway, thereby inhibiting the function of effector T cells, enhancing the stability and immunosuppressive ability of regulatory T cells, creating an immunosuppressive microenvironment conducive to tumor growth, and making cold tumors easier to escape immune surveillance and attack [50]. D. Nucleotide Metabolism. The rapid proliferation of cold tumors necessitates substantial quantities of nucleotides for DNA and RNA synthesis; thus, their nucleotide synthesis pathways are typically highly active [51]. Tumor cells supply the essential material for cell proliferation by enhancing nucleotide synthesis pathways. In the tumor microenvironment, the activation and proliferation of immune cells also consume a significant amount of nucleotides. Consequently, the nucleotide metabolism of tumor cells may be regulated by factors secreted by immune cells, potentially leading to a relatively “normal” nucleotide metabolism. Certain nucleotide metabolism products play immunomodulatory roles within the tumor microenvironment. For instance, adenosine accumulates in the cold tumor microenvironment and binds to adenosine receptors on immune cell surfaces, inhibiting their activity and thereby promoting immune tolerance [52]. Adenosine levels in hot tumors are relatively low, and their immunosuppressive effects are weak.

The application of metabolomics techniques, including nuclear magnetic resonance spectroscopy and mass spectrometry, has revealed significant differences in the metabolite profiles of cold tumors compared to hot tumors. In addition to metabolites from glycolysis, fatty acid metabolism, amino acid metabolism, and other pathways, cold tumors also contain specific metabolites related to tumor proliferation and invasion, such as matrix metalloproteinases (MMPs) and microRNAs (miRNAs). Among these specific metabolites, miR-21 is highly expressed in various cold tumors. It can regulate several biological behaviors, including cell proliferation, apoptosis, and invasion, and by binding to target genes, it inhibits their expression, thereby promoting tumor development [53,54]. Metabolites associated with immune activation and inflammation, such as IFN-γ and IL-6, were highly enriched in hot tumors. Metabolic pathway analysis revealed that cold tumors were primarily enriched in pathways closely related to tumor cell proliferation and survival, including glycolysis/gluconeogenesis, fatty acid synthesis, and amino acid biosynthesis. Conversely, hot tumors exhibited greater enrichment in pathways related to immune response, such as purine metabolism, unsaturated fatty acid metabolism, and amino acid, sugar, and ribose metabolism.

These metabolic abnormalities not only provide the energy and material foundation for the growth and proliferation of tumor cells but also further impair the anti-tumor immune response by affecting the metabolic state and function of immune cells. Through metabolomics studies, metabolic markers associated with cold tumor formation and immunotherapy efficacy can be identified, offering a new perspective for the development of personalized treatment strategies.

#### 2.2.3 Multidimensional Omics and Cold Tumors

Multidimensional omics refers to a research approach that integrates multi-level data, such as genomics, transcriptomics, proteomics, and metabolomics, to systematically analyze biological characteristics [55]. With the continuous advancement of high-throughput sequencing technology and bioinformatics, multidimensional omics research has garnered increasing attention in the field of oncology. This approach can comprehensively unveil the molecular mechanisms underlying tumorigenesis and development. In the field of cold tumor research, multi-dimensional omics analysis plays a crucial role in uncovering key molecules and signaling pathways associated with tumor immune escape, the development of immunosuppressive microenvironments, and the efficacy of immunotherapy. By integrating multi-dimensional omics data, researchers can construct a more precise molecular classification system for cold tumors, thus offering a robust scientific foundation for the formulation of personalized treatment strategies. In addition to the metabolomic approaches mentioned above, the application of genomics can also assist us in identifying mutations in tumor driver genes [55]. Utilizing advanced technologies such as whole-genome sequencing or targeted sequencing, we are able to uncover key gene mutations in cold tumors. For instance, in breast cancer studies, certain cold tumors have been found to harbor specific genetic mutations that may cause tumor cells to evade effective recognition and attack by the immune system [56]. Genomics can also uncover genetic differences within a cold tumor, or between various tumor cell clones, known as tumor heterogeneity. This heterogeneity might result in varied responses of tumors to immunotherapy, potentially influencing the overall treatment efficacy [57].

Transcriptomics enables the analysis of immune-related gene expression. Utilizing transcriptome sequencing technology, we can identify the expression levels of various immune-related genes in cold tumors, encompassing cytokines, chemokines, immune checkpoint molecules, and other categories. The low or high expression of specific immune-related genes may be closely associated with the immunosuppressive state of cold tumors, offering significant clues for the exploration of potential immunotherapy targets [58]. Additionally, transcriptomics can uncover novel immune cell subsets and molecular markers, aiding in the identification of unique immune cell subsets or molecular markers in cold tumors. These discoveries are crucial for a deeper comprehension of the complexity within the cold tumor immune microenvironment and offer a scientific foundation for the development of innovative immunotherapy strategies [59].

Proteomic techniques, such as mass spectrometry, facilitate the identification of immunomodulatory proteins. Through these methods, researchers can uncover the expression patterns of proteins in cold tumor tissues and their microenvironment, including immunomodulatory and signaling pathway-related proteins. These proteins are crucial for the activation, proliferation, migration, and functional regulation of immune cells. Abnormal expression levels of these proteins may be closely associated with the formation and maintenance of the cold tumor immune microenvironment [60]. By analyzing protein-protein interactions and constructing a network of these interactions, we can uncover the complex signal transduction pathways between immune cells and tumor cells in cold tumors, as well as the synergistic or antagonistic effects among immune cells. This provides a foundation for identifying key therapeutic targets and intervention pathways [60].

Single-cell omics technology enables the analysis of immune cell heterogeneity. Utilizing single-cell transcriptome sequencing, we have been able to analyze each cell in cold tumors with high precision, thereby revealing the heterogeneity and fine subpopulation classification of TME cells [61]. This offers insight into the functional status, differentiation processes, and interactions of various cell subsets within the cold tumor microenvironment, as well as their mechanisms of action in immune escape and tumor progression. Utilizing single-cell omics allows for the monitoring of dynamic changes in immune cells during the onset, development, and treatment of cold tumors in real-time, including alterations in cell numbers, subset proportions, functional statuses, and other aspects. This provides a crucial foundation for evaluating the efficacy of immunotherapy, predicting disease progression, and formulating personalized treatment strategies [62].

Spatial transcriptomics enables the mapping of the spatial distribution of immune cells within tissues, allowing researchers to simultaneously capture both the spatial location information of cells and gene expression data on tissue sections. This capability enables investigators to visually reveal the spatial distribution characteristics of immune cells in cold tumors. For instance, studies have identified that the distribution of immune cells in cold tumors is heterogeneous, with lower infiltration of immune cells in certain areas. Additionally, while there may be clusters of immune cells in other areas, these cells could be functionally suppressed [63]. This spatial distribution information is crucial for understanding the interaction between immune cells and tumor cells and for enhancing the precision of immunotherapy. The application of spatial transcriptomics helps to reveal the spatial association and interaction network among various cell types in the cold tumor microenvironment, including proximity effects and signal transmission between immune cells and tumor cells, stromal cells, vascular endothelial cells, etc. By constructing a tumor microenvironment map with high spatial resolution, the biological characteristics of cold tumors can be more deeply understood, and more accurate targets and basis for the development of targeted treatment strategies can be provided [64].

Multidimensional omics research will generate massive data, and its analysis process is extremely complex. Artificial intelligence-based data integration and analysis is undoubtedly a key development direction in the future research field. Through the use of machine learning algorithms, including support vector machine (SVM), random forest (RF), and deep learning, multi-omics data are trained and learned to construct prediction models. This enables a detailed analysis of the mechanisms of cold tumors and the prediction of their clinical outcomes and treatment responses. For instance, employing deep learning algorithms for feature learning and model building on integrated multi-omics data can enhance the diagnostic accuracy and prognosis prediction capabilities for cold tumors [65]. Utilizing the theories and methodologies of network pharmacology and systems biology, researchers have developed a multi-omics network model for cold tumors. This model encompasses a gene-protein-metabolite interaction network and a signaling pathway network. Through network analysis and simulation techniques, researchers have pinpointed key nodes and modules, offering novel insights for the discovery of therapeutic targets and the development of drugs for cold tumors [66].

In conclusion, by integrating multi-omics data for analysis, we can comprehensively delineate the characteristics of cold tumors. The integration of genomics, transcriptomics, proteomics, and other omics data can help to deeply describe the molecular characteristics, immune microenvironment characteristics, and biological behavior of cold tumors from multiple dimensions, and then construct a more comprehensive and accurate cold tumor disease model. This will lead to a deeper understanding of the pathogenesis of cold tumors, immune escape strategies, and mechanisms of response to therapy. The integrated analysis of multi-omics data can systematically reveal potential therapeutic targets and diagnostic markers, which may cover multiple levels such as genes, proteins, metabolism, and are closely related to the immune microenvironment and pathophysiological processes of cold tumors. This provides richer resources and more precise directions for the development of new therapeutic drugs and diagnostic methods. For summary information on multiomics in the analysis of different types of cold tumor studies, refer to Table 2.

**Table 2:** Summary of partial research achievements on cold tumors using multi-omics approaches

Research on diseases	Multidimensional omics technology	Key findings	Insights into cold tumors	References
Cervical cancer	Whole exome, transcriptome, and proteome integrated analysis	Cervical cancer is divided into three subtypes, with the third subtype showing upregulation of complement/coagulation cascade-related proteins and the highest immune score, potentially benefiting from immunotherapy.	Provide molecular classification basis for remodeling the immune microenvironment of cold tumors, suggesting that specific subtypes may transform into ‘hot tumors’ through immune activation.	[67]
Breast cancer	Genome, transcriptome, proteome, metabolome, and imaging integration	The basal-like subtype (Basal-like) has higher levels of lipid peroxidation and expression of ferroptosis-related proteins; immune cell enrichment is found in the hormone receptor positive/HER^2^ negative subgroup.	Expand the population benefiting from treatment through metabolic reprogramming (such as targeting ferroptosis) or immune checkpoint inhibitors, improving the immunosuppressive state of cold tumors.	[68]
Head and Neck Squamous Cell Carcinoma	Transcriptome, proteome, and lipidome joint analysis	OTUB^2^ activates CALML3 through STAT1, promoting phosphatidylserine (PS) synthesis and inhibiting tumor development; oral PS can enhance chemosensitivity.	Metabolic interventions (such as PS supplementation) may reverse the immune escape mechanism of cold tumors, enhancing treatment effects.	[69]
Medulloblastoma	Single-cell RNA/ATAC sequencing, spatial transcriptomics, whole-genome sequencing	Large-scale chromosomal aberrations are tumor-initiating events, oncogene aberrations (such as MYC amplification) are subclonal events, and the dominant clone leads during recurrence.	Suggest that early intervention for cold tumors should focus on chromosomal instability, rather than relying solely on oncogene targeted therapy.	[70]
Pan-cancer angiogenesis	Single-cell transcriptomics, spatial transcriptomics (31 types of cancer)	Tumor angiogenesis originates from venous endothelial cells (VenECs), APLN+ TipSI cells co-localize with an immunosuppressive microenvironment, promoting Treg cell infiltration.	The interaction between the vascular system and the immunosuppressive microenvironment may be a key mechanism in the formation of cold tumors, with anti-angiogenesis therapy potentially synergizing with immunotherapy.	[71]
Metabolomics technology	Four-dimensional metabolomics (Met4DX)	Precisely identify metabolite isomers (such as CCS differences of 1% metabolites), and construct the most comprehensive four-dimensional metabolite database.	Reveal the unique metabolic characteristics of cold tumors (such as hypoxia-related metabolic pathways), providing data support for metabolic targeted therapy.	[72]

Note: With the comprehensive analysis of multi-omics, the research on cold tumors has shifted from a single-mechanism perspective to one of systems biology, which opens up a new avenue for precision diagnosis and treatment. In the future, further integration of spatial omics with clinical data will be necessary to verify the general principles across cancer types.

## 3 Common Strategies for Converting Cold Tumors to Hot Tumors

In clinical treatment, transforming cold tumors into hot tumors is an important strategy for enhancing the response rate of immunotherapy. This transformation boosts the immune system’s ability to recognize and attack tumors, significantly improving the efficacy of immunotherapy. It also expands the applicable population of immunotherapy, potentially prolonging patients’ survival time. Some patients may even achieve long-term control or complete remission of tumors, which can significantly improve their prognosis. Previous research indicates that transforming a cold tumor into a hot tumor presents numerous challenges [44]. These common challenges are summarized in Table 3. In light of these challenges, strategies commonly employed in clinical management are detailed below.

**Table 3:** Major challenges in transforming cold tumors into hot tumors

Challenge field	Specific challenges	Description	Impact
Tumor microenvironment (TME)	Insufficient immune cell infiltration	In cold tumors, there is a lack of immune cells such as cytotoxic T lymphocytes (CTLs), making it difficult to recognize and attack tumor cells.	Immunotherapy (such as PD-1 inhibitors) is ineffective, and the tumor continues to grow.
	Stromal barrier	A dense extracellular matrix (ECM) and CAFs form a physical barrier that hinders the infiltration of immune cells.	Drugs and immune cells cannot effectively reach the core area of the tumor, weakening the treatment effect.
	Hypoxia and acidic environment	Low oxygen and high lactic levels in the TME inhibit immune cell function and promote the aggregation of immunosuppressive cells.	Inhibition of T cell activity, enhancing immune escape.
Immune suppression mechanisms	Immunosuppressive cells	Suppressive cells such as Treg and MDSC secrete anti-inflammatory factors (e.g., IL-10, TGF-β), suppressing the immune response.	Weakening the anti-tumor activity of CTLs, maintaining an immunosuppressive environment.
Immune suppression mechanisms	Overexpression of immune checkpoint molecules	Molecules such as PD-L1 and CTLA-4 are highly expressed in tumor cells or the TME, leading to T cell exhaustion.	Immune checkpoint inhibitors may only be effective for some patients and need to be combined with other therapies.
Tumor heterogeneity	Low tumor antigenicity	Low TMB or insufficient expression of neoantigens prevents the immune system from effectively recognizing the tumor.	Vaccines or T cell therapies have difficulty activating specific immune responses.
Tumor heterogeneity	Clonal heterogeneity	Tumor cell subpopulations have different genetic and phenotypic characteristics, evading immune surveillance.	Single therapy cannot clear all subgroups, easy to relapse.
Technical limitations	Low targeted delivery efficiency	Drugs or gene-editing tools (e.g., CRISPR) have difficulty precisely targeting tumor tissue, posing a high risk of systemic toxicity.	The therapeutic window is narrow, the efficacy is limited and side effects increase.
Technical limitations	Lack of biomarkers	There is a lack of reliable biomarkers to predict which cold tumors may convert to hot tumors or to monitor treatment responses.	Clinical decision-making relies on trial and error, delaying the optimal treatment time.
Clinical translation challenges	Limitations of animal models	Preclinical models (e.g., mice) cannot fully simulate the complexity of the human TME, leading to difficulties in extrapolating data.	Drugs have a high failure rate in clinical trials, increasing research and development costs.
Clinical translation challenges	Complexity of combination therapy	Combining radiotherapy, chemotherapy, and immunotherapy is necessary, but managing doses, timing, and toxicity is complex.	Optimizing the plan is time-consuming, may cause cumulative toxicity, and reduce patient tolerance.
Metabolism and Epigenetic regulation	Metabolic competition	Tumor cells suppress T cell metabolism and function by consuming nutrients such as glucose and amino acids.	T cell proliferation and effector function are impaired, weakening the immune response.
Metabolism and Epigenetic regulation	Epigenetic silencing	Epigenetic modifications of tumor-related genes (e.g., antigen-presenting genes) lead to immune escape.	The immune system cannot recognize tumor cells, increasing treatment resistance.

### 3.1 Adjuvant Radiotherapy and Chemotherapy

Adjuvant radiotherapy and chemotherapy are traditional methods used to convert a cold tumor into a hot tumor. Radiotherapy can directly kill tumor cells, destroy the tumor structure and microenvironment, release tumor antigens, and induce immunogenic cell death (ICD), thereby attracting immune cells to infiltrate the tumor site [73]. Chemotherapy drugs also have similar effects, by killing tumor cells, clearing immune barriers, altering the cytokine profile in the tumor microenvironment, reducing the number of immunosuppressive cells, enhancing the activity of immune cells, and promoting the transformation of cold tumors into hot tumors [74] For instance, it decreases the expression of the immunosuppressive cytokine TGF-β and increases the levels of proinflammatory cytokines Tumor Necrosis Factor-α (TNF-α) and Interleukin-12 (IL-12) [74]. It also reduces immunosuppressive cells in the TME, such as MDSCs and Tregs [75]; thereby enhancing immune cell infiltration and activity. Certain chemotherapy regimens, for example, can promote the infiltration of CD8^+^ T cells and NK cells by reshaping the TME [76]. When combined with immunotherapy, the treatment response rate can be significantly improved, as demonstrated in the treatment of locally advanced rectal cancer (LARC), where neoadjuvant chemoradiotherapy combined with a PD-1 inhibitor markedly increased the pathological complete response rate [77].

### 3.2 Immune Checkpoint Inhibitors (ICIs)

ICIs block pathways such as PD-1/PD-L1 and CTLA-4/B7, thereby lifting the suppression on T cells and allowing them to better recognize and destroy tumor cells [78]. The interaction between PD-1 and its ligand PD-L1 leads to T cell exhaustion and hampers anti-tumor immunity. By inhibiting the PD-1/PD-L1 pathway, ICIs can mitigate this suppression and boost the activity and tumor-killing capabilities of T cells. Research has indicated that high expression of PD-L1 on tumor cells correlates with the effectiveness of ICIs [79,80]. CTLA-4 suppresses T cell activation and proliferation by binding to B7 proteins (CD80 and CD86). Antibodies against CTLA-4, like ipilimumab, can counteract this suppression, thereby bolstering T cell-mediated anti-tumor responses [78]. Other inhibitory immune checkpoint proteins, including Lymphocyte-activation gene 3 (LAG-3), T cell immunoglobulin and mucin domain-containing protein 3 (TIM-3), B and T lymphocyte attenuator (BTLA), and V-domain Ig suppressor of T cell activation (VISTA), are also key targets for the development of ICIs. Compared with hot tumors, cold tumors usually show more obvious ICI resistance. The main mechanism involves the inhibitory characteristics of the tumor immune microenvironment, which prevents effector T cells from effectively infiltrating or activating, achieving deep “immune escape”. There have been relatively detailed review reports on its specific mechanism [81,82]. Studies suggest that different ICIs or their combination with other therapies that stimulate the immune response can effectively activate cold tumors. For instance, the combined administration of anti-PD-1/PD-L1 and anti-CTLA-4 antibodies can further amplify the immune response. This combination therapy strategy markedly enhances treatment outcomes by concurrently blocking multiple immune suppressive pathways. In clinical settings, the combination therapy of ipilimumab (anti-CTLA-4) and nivolumab (anti-PD-1) has demonstrated higher response rates across various types of cancer [78]. Chemotherapy-induced ICD can be used in conjunction with nivolumab to bolster the anti-tumor capabilities of the immune system [74]. HDAC inhibitors (such as Entinostat and Vorinostat) can enhance the efficacy of ICI by inhibiting immunosuppressive cells (such as MDSCs and Tregs) or upregulating antigen-presenting molecules (such as MHC-I), thereby reversing drug resistance. For instance, Entinostat combined with Pembrolizumab was effective in a subset of melanoma and NSCLC patients who failed PD-1 monotherapy in the ENCORE 601 trial, and it was well tolerated [82]. STING agonists (such as STING-LNP) can enhance anti-tumor immune responses by activating APCs, CD8^+^ T cells, and DCs, and can work synergistically with PD-1 blockers to overcome drug resistance (such as in the B16-F10 lung metastasis model). Additionally, the MUSIC platform uses ultrasound-guided STING agonists to target APCs, making low immunogenic tumors sensitive to PD-1 inhibitors [83]. ICIs have been extensively used in treating various cancers, such as melanoma, lung cancer, kidney cancer, and colorectal cancer. These drugs function by alleviating immune suppression and enhancing the anti-tumor activity of T cells, effectively activating cold tumors and significantly improving patient survival rates [77,78].

### 3.3 Tumor Vaccines and Adoptive Cell Transfer Immunotherapy

#### 3.3.1 Tumor Vaccine

Tumor vaccine technology has shown great potential in theory and research to elicit responses from “cold tumors.” In recent years, remarkable progress has been made at various stages of promoting tumor immune responses, significantly enhancing the effectiveness of tumor vaccine therapy. The innovations cover multiple aspects, including antigen design, antigen presentation mediated by MHC molecules, the activation and maturation of DCs, and the specific activation and expansion of T cells. The details are as follows:

**(1) Innovative Antigen Design and Delivery:** Optimizing antigen design and delivery in novel vaccines can break through the “invisible” barrier of tumors. However, Ma et al. [84] developed a Globo H hexosaccharide antigen via a comprehensive chemical-enzymatic synthesis process. This antigen was effectively conjugated with the non-toxic mutant CRM197 protein, resulting in the creation of the Globo H-CRM197 vaccine. Furthermore, they integrated a modular synthetic adjuvant, 3D-MPL (3-O-deacyl monophosphatidylcholine), into the formulation. This inclusion provided a structurally well-defined and controllable Toll like receptor 4 (TLR4) agonist adjuvant, enhancing the vaccine’s efficacy. This synergistic driving strategy successfully transformed the tumor antigen from a state of “immune escape” to one of “efficient activation”. Fu et al. [85] utilized the *in vivo* immune memory of known antigens to treat tumors. Unlike traditional antigen vaccines, the innovative mRNA vaccine does not directly encode intrinsic tumor antigens. Instead, it reprograms tumor tissue *in situ* to activate existing memory effector T cells. Firstly, an mRNA vaccine was constructed using egg white protein (cOVA) as the model antigen. *In vitro* and *in vivo* experiments demonstrated that the mRNA vaccine could effectively reprogram tumor cells, subsequently inducing effector T cells to specifically recognize and kill tumor cells in the mouse model with established immune memory. The study revealed that the vaccine demonstrated a robust anti-tumor effect in three mouse models of solid tumors—melanoma, breast cancer, and colon cancer—and was able to effectively enhance the infiltration and activation of Teff cells at the tumor sites. Upon combining bulk RNA-seq and single-cell RNA-seq analyses, it was discovered that the vaccine could reconfigure the TME to exhibit inflammatory characteristics and bolster the cytotoxic capabilities of effector memory T cell subsets [85]. Compared to traditional vaccine design strategies, the importance of selecting antigens with high specificity and high immunogenicity (TAA and TSA) has significantly diminished, focusing more on the *in situ* expression ability of immune memory antigens within the tumor. This provides a novel approach to the development of tumor vaccines.

**(2) Promote DC Activation and Enhance Antigen Presentation Efficiency:** The latest technical approaches to DC activation vaccines concentrate on enhancing targeting, immunogenicity, and combined therapeutic efficacy. Vaccines significantly enhance antigen presentation efficiency by directly targeting antigen-presenting cells (APCs) and streamlining the antigen recognition and processing mechanism. Lopez et al. [86] reported a DC-targeted mRNA delivery system that identifies up to 20 novel antigens by sequencing clinical patients’ blood and tumor tissues. These antigens are encoded into two mRNA molecules using a liposome nanoparticle (LNP) formulation designed to passively target APCs, particularly DCs, in lymphoid organs. The system activates DCs via the TLR7/8-mediated type I interferon pathway, thereby enhancing co-stimulatory signals and antigen presentation efficiency. This method efficiently delivers personalized neoantigens, thereby activating a potent and long-lasting T-cell immune response and inducing a new (*de novo*) CD8^+^ and CD4^+^ T-cell response. In some patients, the frequency of antigen-specific TCRs in peripheral blood CD8^+^ T cells can reach as high as 5%–20% [86]. Polyzoidis and Ashkan [87] reported on an autologous DC vaccine that utilizes pulsed DC cells derived from tumor lysates of patients. This method significantly improves the presentation efficiency of MHC class I and II-associated peptides (in a single sample, 4386 MHC class I-related peptides showed at least a 1.5-fold enrichment, while 7224 MHC class II-related peptides achieved at least a 1.5-fold enrichment). Furthermore, the DC vaccine expresses key co-stimulatory molecules such as CD40, CD80, CD86, and CD141, which effectively promote DC-T cell interaction, thereby enhancing antigen presentation and T cell activation. Currently, the project is conducting Phase III clinical trials for newly diagnosed patients [87]. Some nano-adsorbents also significantly improve the cross-presentation efficiency of MHC molecules by enhancing antigen uptake and processing. For instance, metal-organic framework (MOF) nanoparticles can efficiently load antigens and prevent the clearance of apoptotic cancer cells by blocking cellular phagocytosis. This leads to the accumulation of damage-associated molecular patterns (DAMPs) and promotes the presentation of antigen MHC class I molecules, thereby enhancing the anti-tumor immune response [88]. Furthermore, genetically engineered DCs have garnered significant attention, particularly through cytokine-armed dendritic cell progenitors (DCPs). By engineering these cells to express two immunostimulatory cytokines—IL-12 and fms-related tyrosine kinase 3 ligand (FLT3L)—DCPs can activate multiple immune cell types and significantly enhance therapeutic efficacy [89]; Vaccine design that targets specific DC subsets, when used in conjunction with immune checkpoint blockade therapy, can significantly enhance therapeutic efficacy. For instance, the combination of a cDC1 vaccine with PD-1 and CTLA-4 inhibitors can activate CD8^+^ T cells and induce long-lasting immune memory, effectively suppressing tumor recurrence [90].

**(3) Strengthen Personalized Vaccine Research and Development for Tumor-Specific Neoantigens:** Utilizing genomic sequencing technology and artificial intelligence algorithms, vaccines are designed with a focus on new epitopes generated by tumor-specific mutations. These serve as the core targets, allowing for the accurate prediction of their binding affinity with patients’ MHC molecules, thereby achieving individualized adaptation [91]. Braun et al. [91] conducted deep sequencing (with an average tumor sequencing depth of up to 200x) on tumors and normal tissues from each patient with clear cell renal cell carcinoma (RCC) to identify tumor-specific nonsynonymous mutations (SNV/Indel) and expression mutations as potential candidates for new antigen sources. “NetMHCpan 4.0,” an AI-driven algorithm for antigen prediction, forecasts the IC50 binding affinity between mutated peptides and patients’ HLA-A/B/C alleles. Utilizing an immunogenicity scoring model that evaluates various parameters comprehensively, such as peptide-MHC binding stability (NetMHCStabPan), proteasome cleavage (NetChop), TAP transport (NetCTLpan), and gene expression (TPM>10), it identifies the top 1% of high-confidence neoantigens to develop personalized neoantigen vaccines, known as “PCVs”. This study pioneers the establishment of a comprehensive closed-loop system encompassing “whole-genome analysis, AI prediction, and individualized vaccine development” within the field of renal cell carcinoma (RCC). It demonstrates that AI algorithms can accurately identify effective new antigens in tumors characterized by a low mutation load, offering a model for the clinical application transformation. The WT1-DC vaccine, developed by Nagai and Karube [92], utilizes tumor tissue samples from patients. Immunohistochemistry (IHC) confirmed high WT1 protein expression (≥3+) and peripheral blood HLA typing of A*24:02. The vaccine demonstrates high affinity binding to the WT1 peptide 235–243 (CYTWNQMNL), forming an “individualized antigen-target” that enhances the immune response and prolongs survival. The clinical transformation technology core of individualized mRNA cancer vaccines encompasses multiple professional fields, such as tumor immunology and bioinformatics. It includes rapid new epitope prediction based on high-throughput sequencing technology, precise identification of tumor-specific antigens through algorithm models; verification of MHC molecule binding using structural biology and molecular biology methods; and mRNA sequence optimization technology based on RNA chemistry and synthetic biology, which involves adjusting the primary and secondary structures of mRNA to enhance its stability and translation efficiency within cells. The comprehensive application of these technologies enables individualized mRNA cancer vaccines to precisely target a variety of cancer indications, offering cancer patients more personalized and efficient treatment options [93,94].

#### 3.3.2 Adoptive Cell Transfer Therapy (ACT)

ACT involves extracting immune-active cells from the body of a cancer patient, expanding and functionally modifying them *in vitro*. After rigorous identification and qualification, these cells are reintroduced into the patient’s body, aiming to directly eliminate tumor cells or activate the body’s anti-tumor immune response [95]. In recent years, significant progress has been made in the research of activating cold tumors through ACT Therapy. The following will elaborate from four perspectives.

**Tumor Infiltrating Lymphocyte (TIL) Therapy: Breaking through the Immunoinfiltration Bottleneck of Cold Tumors**

TIL therapy entails extracting lymphocytes from a patient’s tumor tissue, expanding them in a laboratory setting, and subsequently reintroducing them into the patient to enhance their ability to target and destroy the tumor. TIL therapy is particularly suitable for activating the immune response against cold tumors due to its natural ability to recognize a variety of tumor antigens. It is the first cell immunotherapy approved for the treatment of solid tumors, including metastatic melanoma. In patients with advanced metastatic melanoma, TIL therapy can achieve a “persistent complete response (CR)” and demonstrates potential curative effects, even for those previously unresponsive to immunotherapy. A randomized controlled trial (involving a Dutch/Danish cohort of 168 patients showed that the TIL group achieved an overall response rate (ORR) of 49% compared to 21% in the Ipilimumab group; a complete response rate (CR) of 20% vs. 7%; and a median progression-free survival (PFS) of 7.2 months vs. 3.1 months. Currently, this therapy has been approved by the FDA for use in patients resistant to immune checkpoint inhibitors, such as anti-PD-1 [96]. Furthermore, TIL therapy has demonstrated significant potential in treating solid tumors, including non-small cell lung cancer, colorectal cancer, and breast cancer [96–98]. Sim et al. [99] completely eliminated the tumor and maintained remission for 35 months by isolating TILs naturally present in the tumor of a metastatic colorectal cancer patient that could recognize the mutant KRAS (KRAS G12D) neoantigen, expanding them *in vitro*, and reinfusing them. According to the latest statistics from the Clinicaltrials.gov database (www.clinicaltrials.gov), as of May 2025, there were 128 clinical trials of TIL anti-tumor therapies in progress or planned to recruit participants worldwide, with 71 having completed recruitment. Additionally, three trials are in phase III, and one is in phase IV.

**Cell Receptor Engineered T Cell Therapy-T Cell Receptor Engineered T Cell Therapy (TCR-T): Precisely Targeting Cold Tumor Antigens**

Scientists are employing gene editing technology to introduce specific TCR genes into patients’ T cells, genes that can recognize specific cancer antigens. This process equips the T cells with the ability to specifically identify and eliminate tumor cells, thereby improving the recognition efficiency of low-expressing tumor antigens. Both TCR-T therapy and Chimeric antigen receptor-T (CAR-T) therapy are integral to ACT, yet they differ significantly. CAR-T cells are engineered by inserting a DNA sequence that encodes a chimeric antigen receptor into the patient’s own T cells. The CAR recognizes antigens via the single-chain variable fragment of an antibody. It can only recognize specific antigens on the surface of tumor cells. Traditional CAR T-cells require a relatively high concentration of target antigens to activate and possess rapid and continuous killing abilities, achieving remarkable results in the field of hematologic tumor treatment [100]. In contrast, TCR-T therapy demonstrates greater potential in combating solid tumors, as these tumors often lack distinct biomarkers on their cell surfaces [101]. TCR-T cells recognize tumor antigen peptides presented by MHC molecules through their T cell receptors (TCRs). These antigens, which may originate from intracellular proteins such as tumor-associated antigens, are processed by MHC class I (or II) molecules and presented on the cell surface. Unlike CAR-T cells, which specifically target surface antigens, TCR-T cells can identify antigens derived from both membrane-bound and intracellular proteins, thereby expanding the range of targets they can attack [102]. TCR-T cells only need a very small amount of target antigens to activate, and are more sensitive to low copy number antigens than CAR-T cells. Although their cytotoxicity is slower, it lasts longer [103,104]. In recent years, TCR-T therapy has made significant progress in technological innovation. Kuilman et al. [105] utilized tumor mutation and T-cell receptor (TCR) repertoire analysis, high-throughput DNA synthesis, and functional genetic screening to identify tumor neoantigen-specific TCRs, including those restricted by HLA class I and class II molecules, from inactive frozen tumor biopsy samples. The identified tumor-specific TCRs were transduced into the patient’s autologous T cells using bicistronic vectors, such as those with P2A-linked TCRα/β. After the quantity and function of these cells were enhanced, they were infused back into the patient for treatment. The breakthrough at the core of this innovative research, particularly applicable to solid tumors where tissue is scarce or the mutational burden is low, marks the beginning of translating previously deemed “infeasible” frozen-biopsy techniques into “personalized TCR-T therapy”. Neoantigen-driven TCR-T therapy offers a promising platform for precision immunotherapy of solid tumors. Nonetheless, optimizing antigen screening, enhancing the hit rate, establishing a standardized GMP process, and conducting larger clinical trials to verify its safety and efficacy are still necessary steps [106]. The development of allogeneic TCR-T cell therapy has also become one of the hotspots of current research. By simultaneously deleting the endogenous TCRα and TCRβ chains and inserting the transgenic TCR into the TRAC locus, the risk of graft-vs.-host disease can be avoided [107]. Combining gene editing technology, by engineering the induction of HLA-I molecule deletion and combining with NK cell inhibition, the host immune system’s rejection of allogeneic T cells can be further reduced, which is expected to achieve universal TCR-T cell therapy [105]. Switchable T cell Antigen Receptor (STAR)-T cell therapy is an innovative chimeric antigen receptor T cell therapy that combines the high affinity of antibodies with the high sensitivity of TCRs, aiming to enhance the recognition and killing ability of tumor cells. Through the carefully designed synthetic TCR and antigen receptor, STAR-T cell therapy can identify and target tumor neoantigens presented by HLA-I without relying on the CD8 co-receptor, thereby achieving a synergistic anti-tumor effect of CD8^+^ and CD4^+^ T cells [108]. Research findings that the combination of TCR-T therapy with PD-1 inhibitors or other therapies is expected to enhance the inhibitory effect of overcoming the cold tumor microenvironment, thus further improving the therapeutic effect of anti-cold tumors [102,109].

According to the latest statistics from the Clinicaltrials.gov database, there are a total of 99 ongoing or planned TCR-T anti-tumor clinical trials worldwide. These trials cover a range of solid tumor types, including but not limited to melanoma, lung cancer, liver cancer, and ovarian cancer. The development of these studies is expected to thoroughly verify the safety and efficacy of TCR-T therapy, and to further explore its potential in combination with other immunotherapies. This could provide new treatment options for patients with difficult-to-treat cancers and offer effective strategies for transforming “cold tumors” into “hot tumors”.

**Memory T Cell Therapy: Enhancing Immune Persistence**

Autologous memory T cell therapy for cancer is an advanced immunotherapy approach that has shown great potential in the field of cancer treatment in recent years. Memory T cells, encompassing central memory T cells (TCMs) and tissue-resident memory T cells (TRM cells), have become an important strategy for enhancing the efficacy of cold tumor treatments due to their long-term persistence, rapid response, and self-renewal capabilities. Particularly, TRM cells can survive for extended periods within tissues or tumor microenvironments, continuously patrol and monitor tumor cells, respond rapidly to antigen stimulation, and enhance the infiltration of immune cells in cold tumors. Fu et al. [110] utilized CRISPR-Cas9 gene editing technology to examine the precise effects of IL-2-induced T cell kinase (ITK) deletion on the therapeutic efficacy of CD19-CAR-T cells. ITK deficiency has been found to decrease the expression of co-inhibitory molecules associated with T-cell exhaustion, including PD-1, TIM-3, LAG-3, TIGIT, and CTLA4. CAR-T cells with an ITK gene knockout exhibit a reduced proportion of exhausted cells and an increased proportion of memory cells in animal models, effectively eliminating tumors and enhancing survival rates [110]. Mai et al. [111] utilized CRISPR-Cas9 RNP electroporation to effectively knock out *Regnase-1* and *Roquin-1*, which are genes that regulate T cell inflammation. T cells with a double knockout (DKO) of *Regnase-1* and *Roquin-1*, when engineered into CAR-T or TCR-T cells, demonstrated a significant increase in central memory T cell (TCM) and stem cell-like memory T cell (TSCM) phenotypes both *in vitro* and *in vivo*. These memory subsets possess greater self-renewal capabilities, such as increased expansion following secondary stimulation, and exhibit prolonged survival. Consequently, they display more potent antitumor activity and achieve more durable efficacy in solid tumor models. As of now, the Clinicaltrials database (www.clinicaltrials.gov) indicates that there are 12 clinical trials involving autologous memory T cells for cancer treatment worldwide, all of which are interventional studies. Currently, these trials are primarily concentrated on Phase I and Phase II early clinical trials and are anticipated to achieve breakthroughs in safety and efficacy evaluations.

**Exploration of Novel Cell Therapy: CAR-NK and CAR-M**

**CAR-NK Therapy:** NK cells are a type of innate immune cell that exhibits broad and unique anti-tumor activity. They possess chemotaxis and can be attracted by specific chemokines in the tumor microenvironment, subsequently infiltrating the tumor tissue [112]. Due to the relatively limited amplification *in vivo*, this precisely reduces the risk of serious adverse reactions such as cytokine release syndrome [113]. They have been genetically modified to express a CAR that specifically recognizes and kills tumor cells expressing the corresponding antigen, eliminating the need for strict HLA matching [114]. In the process of resisting immune checkpoint molecules expressed by tumor cells, due to the relatively small number of inhibitory receptors on the surface of NK cells, CAR-NK cells can still maintain a certain degree of activity even in the immunosuppressive microenvironment, thus showing significant advantages over CAR-T cells [115,116]. Studies have shown that CAR-NK cells can penetrate the immunosuppressive microenvironment of solid tumors and may become a complementary regimen for treating cold tumors [117,118]. In the field of solid tumor treatment, numerous clinical studies have been conducted to assess the feasibility of CAR-NK cell therapy for various types of solid tumors, such as pancreatic cancer, glioblastoma, lung cancer, breast cancer, hepatocellular carcinoma, colorectal cancer, and ovarian cancer [119–121]. Li et al. [120] reported a case study involving the use of Robo1-CAR-NK cells to treat liver metastasis from pancreatic ductal adenocarcinoma (PDAC). During treatment, the pancreatic lesions and liver metastases were controlled within five months. The patient experienced only a transient moderate fever, reaching 38.5°C, and no cytokine release syndrome (CRS) or other serious adverse reactions were observed. Although CAR-NK cell therapy is considered safer than CAR-T cell therapy, it still encounters numerous challenges, including limited durability and long-term efficacy, tumor antigen escape, and issues with manufacturing and scalability. It is essential to further refine the structure of the CAR to minimize off-target effects and enhance efficacy, while also addressing the problem of autophagy that arises from NK cell activation [122]. With the official release of various norms and standards for CAR-NK therapy, the industry will enter a new stage of increased normalization and standardization, indicating the beginning of a new era in the development of CAR-NK therapy.

**CAR-M Therapy.** A large number of TAMs are distributed in cold tumors, exhibiting an immunosuppressive (M2) phenotype, which is one of the key factors in the formation of an immunosuppressive microenvironment. Macrophages engineered with CARs exhibit antigen-dependent cytotoxicity, directly activate phagocytosis, release ROS to kill adjacent antigen-negative or positive tumor cells, and activate T cell responses [123]. Zheng et al. [124] discovered that CAR-M-c-Met significantly inhibited the progression of pancreatic cancer in a mouse model and enhanced the therapeutic effect when combined with chemotherapy drugs. CAR-M activates the TME by secreting proinflammatory cytokines, such as TNF-αand IL-6, promoting the infiltration of CD8^+^ T cells, and inhibiting the immunosuppressive functions of M2 macrophages and Tregs [125]. At the same time, CAR-M can also activate T cells via its antigen presentation function and induce “Epitope Spreading,” thereby enhancing the overall anti-tumor immune response [125]. Early clinical studies conducted by Reiss et al. [125] indicated that circulating tumor DNA (ctDNA) levels temporarily decreased following CAR-M treatment, suggesting systemic immune activation. Currently, research into solid tumor-specific targets for CAR-M technology includes HER2 (associated with breast and gastric cancers), c-MET (related to pancreatic cancer), GPC3 (linked to liver cancer), MSLN (associated with mesothelioma), and other prevalent clinical targets for cold tumors [125]. Although previous studies have shown a bright prospect for CAR-M in treatment, it still faces many challenges, including difficulties in preparation and expansion, unsatisfactory migration and distribution *in vivo*, low transfection efficiency, tumor heterogeneity, uncertainty of efficacy, off-target toxicity, and safety concerns. Innovative dual-target and multifunctional CAR designs could enhance therapeutic efficacy and assist in overcoming tumor heterogeneity, such as targeting HER2 and MSLN, or by integrating costimulatory signaling domains, like CD3ζ and FcεRIγ, to boost macrophage activity and persistence [126]. Since autologous monocyte-derived macrophages are currently the primary cell source, the expansion of these cells *in vitro* presents a significant challenge. Induced pluripotent stem cell (iPSC)-derived macrophages have garnered extensive attention as an alternative approach and strategy, anticipated to enable standardized production and large-scale application [126]. CAR-M therapy is currently in early clinical trials, and its efficacy and safety require further evaluation. Nevertheless, CAR-M therapy transforms the TME of cold tumors, activates T cells, triggers systemic immunity, and offers a breakthrough treatment approach for cold tumors. CAR-M therapy is particularly suitable for treating cold solid tumors that do not respond to traditional immunotherapy.

In summary, the key advancements in the field of cold tumor treatment with adoptive cell therapy are reflected in the gradual overcoming of the immunosuppressive microenvironment through specific cell modifications, the integration of immunomodulatory strategies, and the application of new cell types (such as memory T cells, CAR-NK cells). Looking forward, we anticipate more clinical data to verify its long-term safety and broad applicability.

### 3.4 Antibody Therapy Targeting Immune Suppressive Factors

Antibodies targeting immune suppressive factors within the TME can mitigate the suppressive effects of tumors on the immune system, thereby encouraging the infiltration of immune cells into tumor sites. The TME contains a variety of immune suppressive factors, including but not limited to IL-4, IL-6, IL-10, IL-35, IL-1β, and TGF-β.

IL-4 primarily affects the lineage-specific differentiation of Th2 cells and the regulation of humoral immune responses. Except for basophils and mast cells, IL-4 is predominantly secreted by Th2 cells via autocrine signals [127]. Research has indicated that in mouse models of colon and breast cancer, inhibiting IL-4 can diminish the emergence of immune suppressive M2 phenotypes and MDSCs within specific TME, decrease the number of immune suppressive cells in the TME, and improve tumor-specific CD8^+^ T cell responses, thereby enhancing anti-tumor responses [128]. Dupilumab, an IL-4Rα antagonist approved by the U.S. FDA, impedes IL-4-mediated signaling pathways by blocking the binding of IL-4 to its receptor, thus reducing the production of immune suppressive cells and bolstering anti-tumor immune responses. The combination of Dupilumab with anti-PD-1 monoclonal antibodies has demonstrated significant anti-tumor effects in preclinical studies, particularly in ovarian cancer models, where this dual therapy notably extended the survival of mice [129].

IL-10 is a cytokine with diverse immunoregulatory roles. It triggers the JAK1 and Tyk2 signaling pathways, resulting in the phosphorylation of the STAT3 protein, which in turn suppresses the expression of genes related to MHC molecule synthesis [130]. Furthermore, IL-10 can facilitate the ubiquitination modification of MHC molecules, diminishing their stability on the cell membrane and subsequently downregulating the expression of MHC-I and MHC-II molecules on the surface of cancer cells and APCs, ultimately hindering the activation of cytotoxic T cells [130,131]. IL-10 stimulates the IL-10 receptor (IL-10R) and the STAT3 signaling pathway, promoting the polarization of TAMs towards an immune suppressive M2 phenotype and amplifying the immunosuppressive function of MDSCs [132]. Cetuximab-based IL-10 fusion protein (CmAb-IL10) has been shown to prevent dendritic cell-mediated tumor-infiltrating CD8^+^ T cell apoptosis, demonstrating potent antitumor activity [128]. Other IL-10 inhibitors include the monoclonal antibody Anti-IL-10 (BT-063) [133] and the small molecule inhibitor JTE-607 dihydrochloride [134], among others. The development of IL-10 inhibitors may become a key target for the conversion of cold to hot tumors, with significant clinical implications.

IL-35 is a relatively recently discovered immunosuppressive cytokine belonging to the IL-12 family, composed of two subunits, p35 and EBi3, primarily produced by Tregs and tumor cells [135]. By suppressing the function of immune cells such as CD8^+^ T cells and NK cells, IL-35 reduces their cytotoxic effects on tumor cells. Due to IL-35 inhibiting the function of multiple immune cells, it indirectly leads to a decrease in IFN-γ. IFN-γ is a key antitumor cytokine, and its reduction also suppresses the secretion of other various pro-immune cytokines, weakening the immune system’s inhibition of tumors, thereby promoting tumor growth and metastasis [136]. Antibodies targeting IL-35 can enhance antitumor immune responses by blocking its immunosuppressive effects [128]. Thermo Fisher Scientific has developed recombinant monoclonal antibodies against IL-35, such as 6H15L18 and 6HCLC, which specifically bind to IL-35 and block its interaction with receptors, potentially alleviating its immunosuppressive effects in the TME, thereby enhancing antitumor immune responses [137]. Other inhibitory factors in the TME include TGF-β, VEGF, IL-6, IL-13, IDO, PGE2, and ARG1, among others. By targeting these cytokines, immunosuppression can be reduced, immune cell infiltration can be enhanced, and the conversion of cold tumors to hot tumors can be promoted, improving the effectiveness of immunotherapy. This will not be discussed in detail here. The development of cytokine inhibitors in the realm of cold tumor treatment remains in the research phase, primarily concentrating on the modulation of interleukins and several critical immunosuppressive factors. Concurrently, precise intervention via emerging delivery technologies, such as nanoparticles and hydrogels, is also under investigation. Further studies are necessary to confirm their efficacy in counteracting the immunosuppressive microenvironment and to enhance therapeutic outcomes by integrating them with combination immunotherapies, including CAR-T and PD-1 inhibitors.

### 3.5 Targeted Therapy for Abnormal Tumor Vasculature

**Figure 3:**
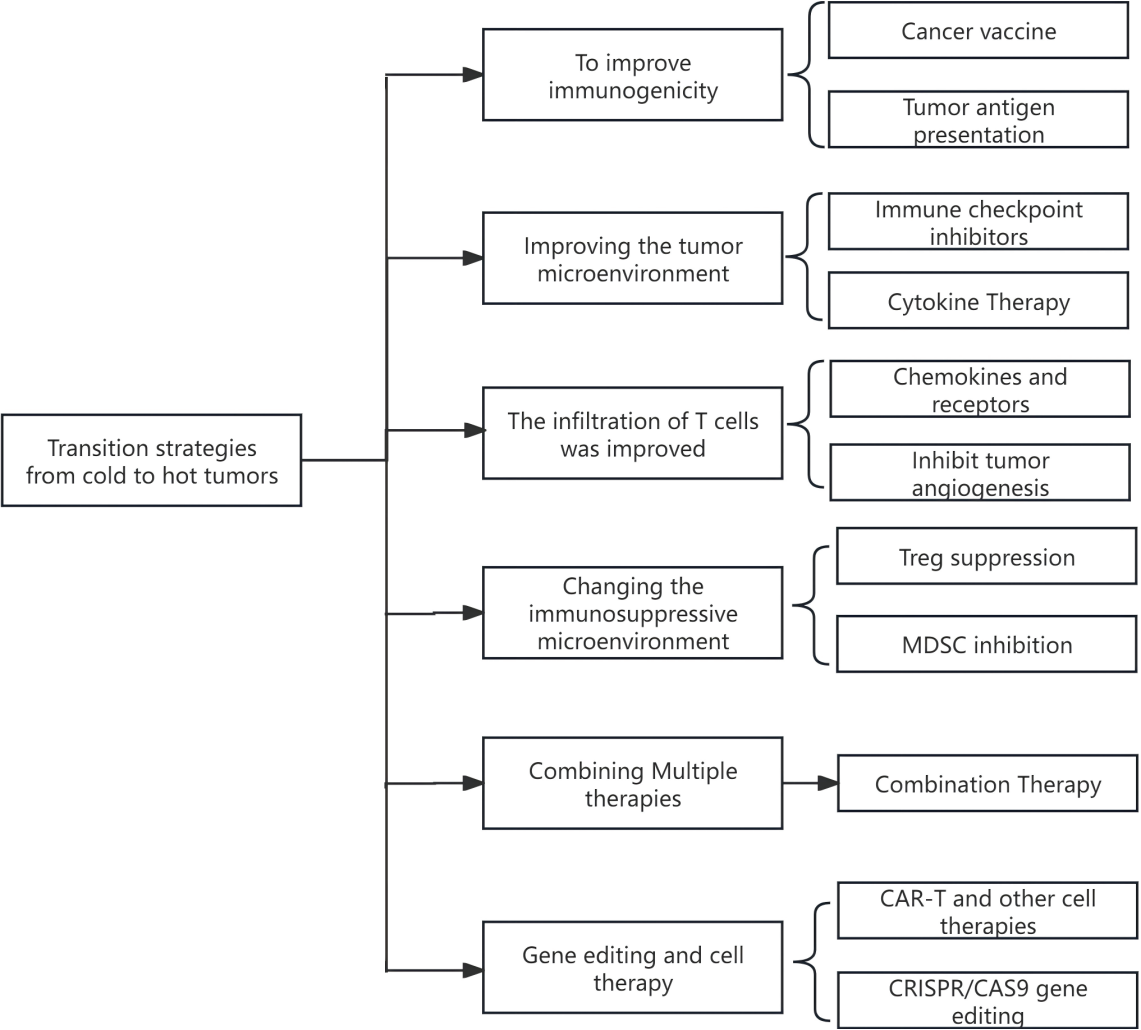
Summary of strategies for the transformation from cold to hot tumors

In the field of tumor vascular therapy research, studies targeting angiogenic signaling pathways and the development of inhibitors have become a core focus. The VEGF/VEGFR pathway is a widely studied and crucial target. Current research is focused on developing more efficient and selective inhibitors of VEGF and its receptors. VEGFR-2 inhibitors with multi-target capabilities are considered promising and highly effective anti-cancer drugs in cancer treatment [138]. For example, tivozanib has IC50 values of 0.21, 0.16, and 0.24 nmol/L for VEGFR-1, VEGFR-2, and VEGFR-3, respectively, and has shown anti-tumor activity in various cancers, including lung, breast, colon, ovarian, pancreatic, and prostate cancers. Its high selectivity results in significantly lower off-target effects than sorafenib, and its half-life is significantly longer than that of sorafenib, allowing for once-daily dosing and no interaction with CYP3A4 inhibitors, with no associated hepatotoxicity or tissue accumulation [139]. Other signaling pathways related to angiogenesis, such as the FGF and its receptor (FGFR) pathway, are another research focus. Studies have revealed that the FGF-FGFR signaling pathway can interact with the VEGF-VEGFR signaling pathway, playing a key role in tumor vascular growth and remodeling. New inhibitors targeting FGFR are currently being developed and tested in preclinical research and clinical trials for evaluation [140]. The Notch signaling pathway plays a critical role in angiogenesis and the construction of tumor vascular networks. Current research is dedicated to deeply analyzing the complex interactions between Notch and other angiogenic pathways, and on this basis, exploring potential therapeutic targets [140]. Additionally, the study has identified other factors related to angiogenesis [141], such as WNT ligands. Blocking the WNT signaling pathway mediated by WNT ligands produced by endothelial cells may help suppress the growth of tumor blood vessels and tumor metastasis [142]. In addition to inhibiting the abnormal generation of tumor blood vessels, the normalization of tumor vasculature is also a key direction in cancer treatment research. Researchers are exploring how specific drugs can achieve tumor vascular normalization by adjusting the balance of factors that promote and inhibit angiogenesis in the TME [143]. At the same time, the role of pericytes in the process of vascular normalization is also receiving a lot of attention. Pericytes are wall cells closely connected with endothelial cells, which have a significant impact on the stability and function of blood vessels. Studies have shown that targeting pericyte-related signaling pathways, such as the β3-integrin expressed by pericytes, a molecule that regulates angiogenesis and stability, may play an important role in promoting vascular normalization and controlling tumor growth [144,145]. The concept of vascular normalization is being applied to enhance the effectiveness of other cancer treatments [146]. For example, by normalizing tumor blood vessels, the delivery efficiency of chemotherapeutic drugs and immunotherapeutic agents to tumor sites can be improved, thus achieving better treatment outcomes [147]. Researchers are evaluating the efficacy of combining drugs that induce vascular normalization with standard cancer treatments in different types of cancer [147,148]. Based on the aforementioned analysis, the strategy of converting cold tumors into hot tumors has shown significant advantages in both preclinical research and clinical treatment, significantly enhancing the efficacy of tumor immunotherapy. This strategy notably strengthens the immune system’s ability to recognize and attack cancer cells, improves the effectiveness of immune checkpoint inhibitors, and enhances the prognosis for patients, increasing the likelihood of longer survival times and higher chances of tumor regression or complete remission. Furthermore, it has also increased the potential for clinical combination therapy. For a summary chart of the strategies for the transformation of tumors from cold to hot, please refer to Fig. 3. Despite the many potential clinical benefits of this strategy, three challenges associated with it should be highlighted: (1) Precisely controlling the degree and scope of immune activation is an extremely challenging task. How can a dynamic balance be found between stimulating tumor immune responses and avoiding autoimmune toxicity? Excessive activation of the immune system in the short term may lead to serious immune-related adverse events (irAEs), such as cytokine release syndrome, pneumonia, and colitis. However, long-term immune intervention may lead to dynamic adaptation of the TME to treatment pressures, resulting in resistance and fluctuations in clinical treatment outcomes. Although researchers continue to strive, implementing individualized precision treatment, immune regulation, and timely monitoring and management strategies have alleviated the contradiction between tumor immune activation and autoimmune toxicity to some extent, the potential risks still exist. Therefore, achieving a balance between pursuing effective treatment and ensuring patient safety and well-being is a significant challenge. It is crucial to accurately identify the time frame for vascular normalization. (2) It is essential to precisely grasp the critical time window for vascular normalization. Anti-angiogenic drugs, such as bevacizumab, can temporarily enhance T cell infiltration and synergistically boost the efficacy of immunotherapy when they induce their pharmacological effect of vascular normalization. However, prolonged use may result in excessive pruning of tumor vasculature, thereby worsening the hypoxic state of the microenvironment [149], which increases the unpredictability of clinical treatment outcomes. Consequently, timely evaluation and monitoring of treatment responses to achieve the optimal medication regimen is essential. Nonetheless, with existing technological capabilities, real-time assessment of alterations in the immune microenvironment during treatment is still difficult, and considering both patient compliance and the cost of analytical technology, it currently lacks feasibility. (3) The heterogeneity and adaptive resistance characteristics of tumors present unpredictable challenges to the technology of converting cold tumors into hot tumors, significantly impacting the effectiveness of immunotherapy. Due to the spatial heterogeneity within tumors, certain areas consistently exhibit “immune deserts,” where immune cells struggle to penetrate. The immune microenvironment within these heterogeneous areas cannot be easily reshaped, which reduces treatment responses and increases the risk of recurrence, significantly hindering the conversion of cold tumors into hot tumors. Furthermore, during the process of managing stress, tumor cells may undergo clonal evolution or a significant emergence of non-dominant, heterogeneous tumor cells. This can lead to the loss of antigens or the upregulation of immune suppressive molecules, such as VISTA, in newly amplified tumor cells. Consequently, new immune escape mechanisms are formed, resulting in treatment tolerance and ultimately causing treatment regimens to fail. Of course, the challenges of converting a cold tumor into a hot tumor in clinical treatment encompass many other aspects, as detailed in Table 3, and will not be discussed here. These unpredictable challenges significantly affect the clinical efficacy of activating cold tumors, urgently requiring more innovative technological approaches to break through the current bottlenecks.

## 4 Future Development Direction

The essence of “activating cold tumors” immunotherapy is to convert those “cold tumors,” characterized by a lack of immune cell infiltration and a significant immunosuppressive microenvironment, into “hot tumors” with heightened immune activity. This transformation aims to enhance the response rate of immunotherapy. Considering the rapid advancements in biotechnology, we can anticipate several promising future development paths and research strategies. Subsequently, this paper will provide a brief analysis of some of the more mature technologies and their potential future development trends.

### 4.1 Innovative Technology Targeting the Immune Suppressive Microenvironment

#### 4.1.1 Proteolysis-Targeting Chimeras (PROTACs) Technology Breakthrough for Undruggable Targets

Since its inception in 2001, the concept of PROTAC molecules has garnered widespread interest from both academic and industrial sectors. With ongoing advancements in PROTAC design and thorough evaluation in preclinical models, the first cohort of PROTAC molecules, ARV-110 and ARV-471, entered the clinical research phase in 2019. Presently, nearly 40 PROTAC molecules globally have been approved for clinical trials, with indications ranging from cancers to autoimmune diseases, among others. In numerous studies, the PROTAC molecule ARV-471 entered the clinical phase III trial stage at the end of 2022 [150]. According to the information provided by Clinicaltrials, ARV-471 is currently under in-depth study in 12 clinical trials for breast cancer, marking a new approach in the exploration of cancer treatment. PROTAC technology represents an innovative therapeutic approach that utilizes the cell’s own protein degradation mechanism—the Ubiquitin-Proteasome System (UPS)—to selectively degrade specific proteins associated with diseases through an “event-driven” approach. This may enable the modulation of “undruggable” targets that are challenging for traditional small molecule drugs to affect. Taking cold tumors as an example, the expression of HLA-I is suppressed due to epigenetic silencing (such as DNA methylation), which leads to a defect in neoantigen presentation. However, employing PROTAC technology to degrade epigenetic modification enzymes (such as EZH2) can restore antigen presentation function [150]. By designing delivery systems like nanoparticles and prodrug strategies, the solubility, targeting, and bioavailability of PROTAC can be improved, thereby reshaping the immune microenvironment of cold tumors [151]. Despite the potential of PROTACs in treating diseases such as cancer, their clinical development is constrained by challenges in pharmacokinetic optimization, the development of effective biomarkers, and the difficulty in targeting membrane or extracellular proteins. Regarding safety, there is a risk of the “on-target but off-tissue” effect, where the effective degradation of the target protein may also affect the same protein in normal tissues, potentially causing systemic toxicity [152]. The main reasons are related to the widespread expression of E3 ubiquitin ligases, the lack of tissue specificity of delivery systems, the amplification of Hook effect in specific tissues, and the non-specificity of target proteins [152]. In terms of their formulations, PROTACs typically have a large molecular weight (>700 Da), strong hydrophobicity, and high polarity. These characteristics result in poor water solubility, weak cell permeability, and suboptimal pharmacokinetic properties, which in turn limit their oral bioavailability and tissue permeability [153]. Therefore, to overcome these limitations, the structural design of PROTACs, the development of novel delivery systems (such as tissue-specific delivery), and the expansion of the E3 ligase library are necessary for clinical application.

In summary, PROTAC technology has shown great potential in overcoming tumor drug resistance with the ultimate goal of inducing protein degradation. In addition, the technology can also be used for precision treatment of targets that are difficult to become drugs, and effectively regulate the tumor microenvironment, which is expected to bring a revolutionary breakthrough in the treatment of cold tumors.

#### 4.1.2 Novel Nano-Immunomodulators Reshape the Tumor Microenvironment

The term “engineered nanoplatform” refers to a collection of nanomaterials or nanostructures that have been designed and modified by humans specifically for certain biomedical applications. These platforms, which have been innovatively designed and modified, typically possess the following characteristics and functions: (1) Precision targeting. New engineered nanoplatforms can specifically recognize and bind to certain antigens or receptors on the surface of tumor cells through surface modifications, such as antibodies, peptides, and other targeting molecules, achieving precise delivery of drugs to tumor cells [154]. (2) Multi-functional integration. Engineered nanoplatforms can integrate multiple functions, such as drug delivery, diagnostic imaging, and therapy. For example, some nanoplatforms can simultaneously carry chemotherapeutic drugs and imaging agents, realizing the integration of diagnosis and treatment [155]. (3) Responsive design. Engineered nanoplatforms can be designed according to the characteristics of the tumor microenvironment, allowing them to release drugs under specific conditions. For example, some nanoplatforms can release drugs in the acidic environment within tumor cells or under the action of overexpressed glutathione (GSH) [154]. Therefore, nanomedicines based on new engineered nanoplatforms can intervene in the TME through various mechanisms, enhancing anti-tumor immune responses. For instance, by loading immune agonists (such as STING agonists, TLR ligands) or regulating immune suppressive factors (such as TGF-β, IL-10), precise intervention in TME can be achieved [156,157]. Currently, some new engineered nanoplatform anti-tumor drugs have entered the clinical research stage, including: the mRNA vaccine CV9103 designed by CureVac, which contains 4 TAAs and has shown significant efficacy in the treatment of prostate cancer, is currently in the clinical phase II stage [158]; BioNTech has developed a personalized mRNA vaccine BNT122 using liposomal complexes as carriers, which can encode 20 tumor neoantigens, and the treatment for melanoma and colorectal cancer has entered the clinical phase II stage [159]; a supramolecular therapeutic nanoplatform designed by the Chen, H team, based on the “AND” logic gate, is used to overcome resistance to non-small cell lung cancer. This drug delivery system provides a highly controlled and targeted treatment method, effectively inhibiting tumor growth, suppressing bypass signaling pathways, and overcoming EGFR-TKI resistance [160]. Although novel nanoimmunomodulators have shown great potential for converting “cold tumors” into “hot tumors” and enhancing the response to immunotherapy, they still encounter several key limitations during clinical translation. Inorganic nanomaterials, (uch as metal and silicon nanoparticles, or highly immunogenic carriers, may trigger a systemic inflammatory response or be rapidly cleared by macrophages, thereby reducing targeting efficiency [161]. Nanoplatforms coated with cell membranes can fail due to host immune recognition; thus, hybrid membranes, such as those combining red blood cell and cancer cell membranes, are required to reduce immunogenicity [162]. However, scaling up the production of nanocapsules with complex structures, (uch as multi-layer responsive particles, from the laboratory to the clinic presents challenges related to process stability and cost [161]. Additionally, it may involve numerous challenges, such as the absence of biomarkers, the complexity of combination therapy, the unknown pharmacokinetics (PK) and pharmacodynamics (PD), and the regulatory and standardization hurdles. Despite the clinical limitations nano-platforms still face in treating “cold tumors,” their prospects remain generally optimistic. Nano-platforms for treating cold tumors have evolved from a “scientific hypothesis” to “clinically accessible technology,” and their systemic immune remodeling capabilities are anticipated to overcome the bottleneck of traditional immunotherapy.

### 4.2 Exploration of New Targets and New Mechanisms

In recent years, the exploration of new targets and mechanisms for tumor treatment has been in full progress worldwide. Some innovative targets and mechanisms are shown in Table 4.

**Table 4:** Exploration of new targets and mechanisms for tumor therapy

Target/mechanism name	Mechanism of action	Indications	Related therapies/medications	Reference
PILRα Immune checkpoint	PILRα binds to T cell CD99, inhibiting T cell activation; targeted antibodies block this pathway, restoring T cell anti-tumor activity	Glioma, triple-negative breast cancer, etc.	Monoclonal Antibody C21 (Combined with PD-1/CAR-T)	[163]
HCAR1 (lactic receptor)	G protein coupled receptors (GPCRs) family member HCAR1 senses lactic signals driving immune escape; inhibitors block lactic-mediated immune suppression	Various solid tumors	HCAR1 Inhibitor	[164]
Copper death-related genes (CRGs)	Genes such as SLC31A1 regulate copper ion metabolism, affecting glioma cell proliferation and migration; inhibiting CRGs can induce copper death	Glioma	Small molecule inhibitor (e.g., MP-HJ-1b)	[165]
EGFR/Notch dual-target antibody	Simultaneously blocking EGFR and Notch signals inhibits tumor stem cell drug resistance and solid cell proliferation	Non-small cell lung cancer, colon cancer, etc.	CT16 Bispecific antibody	[166]
JAML^+^ CD8^+^ T cell activation	Activating JAML enhances the oxidative phosphorylation pathway of CD8^+^ T cells, improving anti-tumor immune response	Hepatocellular carcinoma (HCC)	Agonistic Anti-JAML antibody	[167]
Nanoparticle-ultrasound combined therapy	Nanoparticles carrying chemotherapeutic drugs, released through ultrasound triggering, enhance tumor mechanical destruction and targeted drug delivery	Melanoma and other solid tumors	Peptide-Modified nanoparticles loaded with chemotherapeutic drugs	[168]
Solanine nanovesicles (SN-NPs)	pH-responsive nanocarriers load solanine, regulating Bax/Bcl-2 and CDH-1/MMP2 genes, inducing apoptosis and inhibiting metastasis	Breast cancer (e.g., MCF-7 cells)	SN-NPs	[169]
Engineered adipocyte metabolic competition	Genetically engineered adipocytes enhance glucose/fatty acid metabolism, competing with tumors for resources, inhibiting growth	Breast cancer, pancreatic cancer, etc.	AMT (Adipose Manipulation Transplantation Technique)	[170]

Note: Innovative immune checkpoint therapies, such as PILRα and HCAR1, regulate immune evasion by T cell inhibition and lactic signaling, respectively, expanding immune therapy targets beyond PD-1/CTLA-4; Metabolic intervention: including cuproptosis, lactic signaling blockade, and fatty acid metabolism competition, treatment is achieved by reprogramming the tumor microenvironment metabolism; Dual-target/multifunctional therapy: for example, CT16 antibody and nanoparticle-ultrasound combined therapy, combining different mechanisms to enhance efficacy and reduce drug resistance; Delivery technology breakthrough: Active drug delivery systems and nanocarrier technologies significantly enhance drug targeting, solving the toxicity issues of traditional chemotherapy.

Bao et al. [171] discovered that Ubiquitin-like modifier activating enzyme 1 (UBA1) plays a crucial role as a mediator of tumor immune responses. The E3 ubiquitin ligase STUB1 facilitates the proteasomal degradation of the essential interferon signaling molecule JAK1, resulting in the suppression of the IFN pathway and the down-regulation of MHC-I and chemokines CXCL9/10, thereby creating an “immune cold” microenvironment. Inhibiting its activity enhances T-cell recruitment and diminishes tumor resistance to immunotherapy. The inhibitor TAK-243 demonstrated rapid and potent anti-leukemic effects in Acute Myeloid Leukemia (AML) models, with minimal impact on normal hematopoietic progenitor cells [172]. Silencing UBA1 also significantly inhibits the growth and migration ability of breast cancer cell lines, such as MCF-7 and MDA-MB-231 [173]. Moreover, at least one UBA1 inhibitor, TAK-243, is currently undergoing clinical trials, offering new possibilities for combined immune checkpoint blockade therapy. Shen et al. [174] discovered that the short form of IL-15 (short IL-18), generated by caspase-3 cleavage in tumor cells, is neither secreted extracellularly nor binds to IL-18Rα. Instead, it enters the nucleus and promotes the phosphorylation of STAT1 at Ser727 via CDK8. Additionally, it enhances the secretion of Interferon-Stimulated Gene 15 (ISG15), thereby mobilizing NK cells to exert anti-tumor functions, such as activating NK cells, increasing their expression of Granzyme B and IFNγ, and significantly inhibiting tumor growth. This effect is independent of the traditional mature IL-18/IL-18R axis. He et al. [164] discovered that HCAR1, a member of the G protein-coupled receptor (GPCR) family, promotes tumor immune escape by sensing lactic signals. They systematically elucidated the potential application value of lactic receptor HCAR1 inhibitors in tumor immunotherapy. Targeting HCAR1 is anticipated to transform “cold tumors” into “hot tumors,” enhance the response rate of immunotherapy, and has significant clinical translational potential by alleviating lactic-mediated immunosuppression, reprogramming the immune microenvironment, and through a synergistic ferroptosis mechanism. Jiang et al. [175] demonstrated that the high expression of TP63 in squamous cell carcinoma is related to the immune cold phenotype. By blocking T cell infiltration through the inhibition of the IFN-γ-STAT1 axis, inhibition of TP63 or activation of the IFNγ signal could reverse immune escape and promote CD8^+^ T cell infiltration. Therefore, TP63 is a targetable driver of “cold tumors,” and its inhibitor or a combined immunotherapy strategy is expected to provide new options for patients with cold tumors that fail to respond to immunotherapy. Intervention studies on the TP63/STAT1/IFNγ axis also provide a theoretical basis for the development of biomarkers and drug combinations. Utilizing CRISPR-based screening, Murayama et al. [176] discovered that inhibiting RNA helicase DHX9 could elevate levels of endogenous nucleic acids, including double-stranded RNA, in cancer cells. This elevation creates a state akin to a viral infection, thereby triggering “endogenous immune” responses such as the interferon response. The novel Viral Mimicry approach has successfully transformed cold tumors by activating endogenous immunity. Furthermore, a range of valuable new therapeutic strategies, including FGFR inhibitors, clinical combination therapy, and H2S/NO-enhanced ICD-mediated tumor therapy, are also implicated [3,177,178]. The newly discovered tumor immunotherapy targets may have potential interdependent or synergistic regulatory effects. For instance, the co-regulation of the TP63-hCAR1 immunosuppressive network could involve lactic-induced HCAR1 signaling activating NF-κB and STAT3, which may indirectly enhance TP63 expression or the stability of its downstream target genes, thereby reinforcing the immune escape program. Conversely, inhibition of TP63 on IFN-γ may also impair DC sensing of lactic and indirectly reduce hCAR1-mediated immunosuppressive feedback. Activation of HCAR1 promotes glycolysis and the accumulation of lactic, induces oxidative stress and protein misfolding, and may activate a ubiquitination stress response mediated by UBA1. Inhibition of UBA1 can result in the collapse of protein homeostasis, thereby increasing the sensitivity of tumor cells to lactic toxicity and creating a metabolic-immune double hit. The TP63-UBA1 transcription and protein homeostasis regulation loop is such that UBA1 inhibition may result in the blockage of TP63 protein degradation, thereby enhancing its immunosuppressive function. However, simultaneously targeting TP63, as with RNA interference, can disrupt this homeostasis and amplify the immunogenic cell death caused by UBA1 inhibition. Although there is no direct evidence that these three pathways form linearly dependent pathways in cold tumors, their functional networks exhibit multiple intersections. The combined targeting of any two or all three is expected to break immune tolerance through a multi-dimensional approach and achieve an effective transformation of “cold-hot” tumors, which warrants further verification in preclinical models.

Research trend analysis indicates that numerous studies have highlighted the synergistic effects of combination drugs, for instance, the pairing of immune checkpoint inhibitors with targeted antibodies, or the integration of physical and chemical therapies, such as the combination of ultrasound with nanomedicine. Treatments tailored to gene expression profiles or metabolic characteristics, like those using AMT technology, and the modification of natural drugs, such as the nano-formulation of solanine, are emerging as research focal points for personalized and low-toxicity treatments. These latest findings provide potential new targets and explanations of mechanisms for cold tumor treatment, with significant clinical implications, and are expected to bring more effective treatment options for patients with cold tumors.

### 4.3 Frontier Technologies and Interdisciplinary Integration

The progress in cold tumor immunotherapy is profoundly integrating cutting-edge global technologies with interdisciplinary innovative thinking, a trend that has become particularly evident in recent years of research. The integration of these technologies not only expands the horizon of treatment but also provides a solid foundation for the development of personalized medical plans, making future cold tumor treatments more precise and efficient.

#### 4.3.1 Breakthrough Applications of Gene Editing and Immune Regulation

Tumor immune escape and drug resistance have long been intractable challenges in clinical treatment. CRISPR technology, by precisely locating and editing specific gene sequences, can identify genes related to the growth, proliferation, differentiation, invasion, and drug resistance of tumor cells, thereby simplifying research processes and efficiently advancing the understanding of tumor molecular mechanisms. The research team at New York University, including Chen, X, etc., revealed through CRISPR-Cas9 whole-genome screening that the SUSD6/TMEM127/WWP2 (STW) axis in leukemia degrades MHC-I through ubiquitination. Targeted inhibition of this axis can restore antigen presentation function and enhance the killing effect of T cells [179]. This discovery has opened new horizons for the application of gene editing technology in studying the mechanisms of cold tumor immune escape, and the formation mechanism of the suppressive microenvironment of solid tumors will be more deeply explained and understood. At the same time, the application of gene editing technology in clinical treatment has also shown unprecedented precision, capable of repairing or regulating specific genetic mutations, thereby reversing the state of immune suppression [180]. This technological breakthrough not only promises to promote the development of cold tumor treatment strategies but may also lead a revolution in the field of tumor treatment. By combining gene editing with immunotherapy, scientists can more precisely locate and intervene in the key links of tumor immune escape, thereby activating the patient’s immune system to more effectively identify and attack tumor cells.

#### 4.3.2 AI and Bioinformatics

The integration of AI with bioinformatics has provided robust support for researchers to deeply understand the immune microenvironment of cold tumors, which helps in uncovering potential immunotherapy targets. Using AI algorithms, researchers are able to conduct in-depth analysis of vast tumor genomic, transcriptomic, and proteomic data, revealing the unique immune escape mechanisms and immune suppression networks of cold tumors. Currently, some preliminary research results have emerged.

stKeep is an innovative heterogeneous graph learning method specifically developed for analyzing spatially resolved transcriptomics (SRT) data to precisely parse the TME. This method captures the complex relationships between cells, genes, and tissue regions through heterogeneous graph learning, integrating multimodal data and gene-gene interactions, and is capable of identifying cellular modules and gene modules within the TME, as well as cell-cell communication (CCC). In various cancer samples, including breast cancer, lung cancer, colorectal cancer, and liver metastatic cancer, stKeep outperforms other tools in accurately identifying cellular states within the TME, such as bipotent basal cell populations, tumorigenic myoepithelial cells, and metastatic cells. Additionally, stKeep can identify key transcription factors, ligands, and receptors related to disease progression, which have been further validated through functional and survival analyses of independent clinical data, highlighting its significant potential in clinical prognosis and immunotherapy applications [181]. Yang et al. [182] constructed the BEPH (BEiT-based model Pre-training on Histopathological images) model, using the BEiTv2 Transformer and employing Masked Image Modeling (MIM) for self-supervised pre-training. The training data scale covers 32 types of cancers, with approximately 11,760 full-field digital slices (WSI), which are divided into 11.77 million 224 × 224-pixel image blocks. The model was tested on 16 public datasets, and BEPH outperformed existing weakly-supervised and specialized models in multiple indicators, with an average improvement of 5%–15%. When the training data volume was reduced to 20%, the performance decreased by less than 3%, demonstrating good small-sample adaptability. The visualization heatmap shows that the model’s focused areas highly overlap with the tumor regions labeled by pathologists, demonstrating interpretability. BEPH achieved “one model for multiple uses” in the diagnosis and survival prediction tasks of 32 types of cancers through “large-scale self-supervised pre-training + lightweight fine-tuning”, significantly reducing data annotation requirements and improving cross-cancer generalization ability. The existing rich genomic, transcriptomic, Metabolomics and proteomic data in the field of bioinformatics provide valuable training materials for AI models, enabling them to more accurately identify tumor-related biomarkers. Therefore, we firmly believe that the deep integration of future AI technology and bioinformatics will certainly pave new ways for understanding the mechanisms of cold tumor development and optimizing immunotherapy strategies. Through deep learning and big data analysis, AI will be able to more accurately predict patients’ responses to immunotherapy, thereby formulating better personalized treatment plans, achieving personalized precision treatment, improving treatment outcomes, and reducing side effects.

#### 4.3.3 The Integration of Nanotechnology and Biotechnology

The integration of nanotechnology and biotechnology has brought revolutionary advances to the field of cold tumor immunotherapy. Through nanomedicine delivery systems, drugs can achieve precise targeting and controlled release, significantly increasing the concentration of drugs in tumor tissues while minimizing damage to healthy tissues. At the same time, the application of biotechnological engineering makes the modification of immune cells possible, endowing them with stronger anti-tumor capabilities and higher safety. The utilization of nanotechnology platforms further promotes the development of synthetic biology. Nanotechnology plays a crucial role in gene regulation, enabling precise temporal and spatial control over gene expression. By combining nanoparticles with gene regulatory elements, the unique physical and chemical properties of nanoparticles, such as optical and magnetic characteristics, can be used to remotely manipulate gene expression. This gene regulation strategy based on nanotechnology provides innovative methods for the construction and regulation of complex gene networks in synthetic biology [183]. In turn, synthetic biology can expand the application scope of nanotechnology. Through techniques such as genetic engineering, synthetic biology can customize the modification of organisms, utilizing their metabolic pathways to synthesize bio-nanoparticles with specific structures and functions. For example, by genetically modifying bacteria, yeast, and other microorganisms, they can be made to produce exosomes, microvesicles, and other bio-nanoparticles that can carry drugs, nucleic acids, and other active ingredients for the treatment and diagnosis of diseases [183]. The fusion of synthetic biology and biomaterials science provides powerful tools for the development of new immunotherapies, such as synthetic biosensors for real-time monitoring of changes in the tumor microenvironment [184], and intelligent responsive materials for the on-demand release of therapeutic molecules [185].

The deep integration of these cutting-edge technologies with interdisciplinary fields will greatly advance the development of cold tumor immunotherapy and herald a new era of personalized precision medicine. In the future, as cutting-edge technologies continue to evolve and interdisciplinary fields become more deeply integrated, the cold tumor immunotherapy field is expected to see the emergence of more innovative technologies and treatment methods, offering increased hope and positive outcomes for patients with cold tumors.

## 5 Summary and Outlook

In recent years, significant progress has been made in the field of cold tumor immunotherapy. Not only has the formation mechanism of cold tumors been more deeply revealed, but a series of effective treatment strategies have been explored. These strategies encompass various aspects, including adjuvant radiochemotherapy, immune checkpoint inhibitors, tumor vaccines, and adoptive immunotherapy of immune cells, providing patients with a diverse range of treatment options. Of particular importance is the integration of cutting-edge technologies and interdisciplinary fields, such as gene editing, artificial intelligence, nanotechnology, and biotechnology, which have brought new technological breakthroughs and potential infinite possibilities to cold tumor immunotherapy. The application of gene editing technology, especially CRISPR-Cas9, has enabled scientists to precisely locate and edit genes related to tumor immune escape and drug resistance, thereby reversing the state of immune suppression and activating the patient’s immune system. The breakthroughs from the integration of this technology with other interdisciplinary fields not only promise to advance “cold tumor” treatment strategies but may also lead to a revolution in the field of tumor treatment. Moreover, the combination of artificial intelligence and bioinformatics has helped researchers gain a deeper understanding of the immune microenvironment of cold tumors, uncover potential immunotherapy targets, and provide strong support for personalized precision treatment. In addition, the integration of nanotechnology and biotechnology has also brought new breakthroughs to cold tumor immunotherapy. Nanomedicine delivery systems can achieve precise drug targeting and controlled release, enhancing treatment efficacy and reducing side effects. The cross-application of synthetic biology and biomaterial sciences has provided powerful tools for the development of new immunotherapies. The deep integration of these cutting-edge technologies will greatly promote the development of the cold tumor immunotherapy field, bringing greater hope and good news to patients.

The development trend of the cold tumor immunotherapy field presents a mix of challenges and opportunities as we look forward. On one hand, the use of innovative technological detection methods allows us to delve deeper into the systemic pathological mechanisms of cold tumor formation and immune escape, discover new signaling regulatory pathways, biomarkers, and drug targets. This provides a solid theoretical foundation for the development of more effective treatment strategies. On the other hand, we must enhance the integration and innovation of cutting-edge technologies with interdisciplinary fields. By combining genetic engineering, targeted protein degradation, nanobiotechnology, and dynamic combination therapy strategies, among other comprehensive approaches, we can promote multi-dimensional regulation of the immune microenvironment. With the continuous integration and advancement of intelligent sensor and deep learning technologies, the realization of self-powered sensing and multimodal signal acquisition technologies has become possible. This opens new avenues for real-time monitoring of the TME in complex environments [186], thereby further advancing the development of personalized precision therapy. As innovative technologies continue to update, future development will require not only the construction and improvement of more efficient medical translation platforms but also the strengthening of international cooperation and exchanges to jointly address the global health challenge of cold tumors. We firmly believe that in the near future, with the continuous progress and innovation of science and technology, the field of cold tumor immunotherapy will surely embrace even more splendid achievements.

## Data Availability

All the information or data in this review are from publicly published international journals.

## Abbreviations

**Abbreviation**: **Full Form**
4-HNE: 4-hydroxynonenal
ACT: Adoptive cell transfer therapy
AI: Artificial intelligence
Ang: Angiogenin
APC: Antigen presenting cell
BTLA: B and T lymphocyte attenuator
CAFs: Tumor associated fibroblasts
CAR: Chimeric antigen receptor
CCC: Cell to cell communication
CHIEF: Clinical histopathology imaging evaluation foundation
CmAb-IL10: Cetuximab-IL10 fusion protein
cOVA: Chicken ovalbumin
ctDNA: Circulating tumor DNA
DAMPs: Damage associated molecular patterns
DC: Dendritic cell
DCPs: Dendritic cell progenitors
FGF: Fibroblast growth factor
FGFR: Fibroblast growth factor receptor
GPCRs: G protein coupled receptors
GSH: Glutathione
HIF: Hypoxia inducible factor
HRE: Hypoxia responsible element
ICD: Immunogenic cell death
ICIs: Immune checkpoint inhibitors
IDO: Indoleamine-2,3-dioxygenase
IFN-γ: Interferon-γ
IL-10: Interleukin-10
IL-10R: IL-10 receptor
IL-12: Interleukin-12
iPSCs: Induced pluripotent stem cells
irAEs: Immune-related adverse events
LAG-3: Lymphocyte activation gene-3
LARC: Locally advanced rectal cancer
LNP: Lipid nanoparticle
MDSCs: Myeloid-derived suppressor cells
MHC: Major histocompatibility complex
MOF: Metal-organic framework
NK cells: Natural killer cells
NRF2: Nuclear factor erythroid 2-related factor 2
NSCLC: Non-small cell lung cancer
PROTACs: Proteolysis-targeting chimeras
PSMA: Prostate specific membrane antigen
ROS: Reactive oxygen species
SRT: Spatially resolved transcriptomics
TAAs: Tumor associated antigens
TAMs: Tumor associated macrophages
TAP: Transporter associated with antigen processing
TCR-T: T cell receptor engineered T cell therapy
Teff: Effector T
TGF-β: Transforming growth factor β
TILs: Tumor infiltrating lymphocytes
TIM-3: T cell immunoglobulin and mucin domain-containing protein-3
TLR: Toll like receptor
TMB: Tumor mutation burden
TME: Tumor microenvironment
TNF-α: Tumor necrosis factor-α
Tregs: Regulatory T cells
TRM cells: Tissue-resident memory T cells
TSA: Tumor specific antigen
UBA1: Ubiquitin-like modifier activating enzyme 1
UPS: Ubiquitin proteasome system
VEGF: Vascular endothelial growth factor
